# Identifying carbohydrate-active enzymes of *Cutaneotrichosporon oleaginosus* using systems biology

**DOI:** 10.1186/s12934-021-01692-2

**Published:** 2021-10-28

**Authors:** Tobias Fuchs, Felix Melcher, Zora Selina Rerop, Jan Lorenzen, Pariya Shaigani, Dania Awad, Martina Haack, Sophia Alice Prem, Mahmoud Masri, Norbert Mehlmer, Thomas B. Brueck

**Affiliations:** Werner Siemens-Chair of Synthetic Biotechnology (WSSB), Technical University of Munich, Lichtenbergstraße 4, 85748 Garching, Germany

**Keywords:** *Cutaneotrichosporon oleaginosus*, Carbohydrate metabolism, Carbohydrate uptake, Proteomics, Protein secretion

## Abstract

**Background:**

The oleaginous yeast *Cutaneotrichosporon oleaginosus* represents one of the most promising microbial platforms for resource-efficient and scalable lipid production, with the capacity to accept a wide range of carbohydrates encapsulated in complex biomass waste or lignocellulosic hydrolysates. Currently, data related to molecular aspects of the metabolic utilisation of oligomeric carbohydrates are sparse. In addition, comprehensive proteomic information for *C. oleaginosus* focusing on carbohydrate metabolism is not available.

**Results:**

In this study, we conducted a systematic analysis of carbohydrate intake and utilisation by *C. oleaginosus* and investigated the influence of different di- and trisaccharide as carbon sources. Changes in the cellular growth and morphology could be observed, depending on the selected carbon source. The greatest changes in morphology were observed in media containing trehalose. A comprehensive proteomic analysis of secreted, cell wall-associated, and cytoplasmatic proteins was performed, which highlighted differences in the composition and quantity of secreted proteins, when grown on different disaccharides. Based on the proteomic data, we performed a relative quantitative analysis of the identified proteins (using glucose as the reference carbon source) and observed carbohydrate-specific protein distributions. When using cellobiose or lactose as the carbon source, we detected three- and five-fold higher diversity in terms of the respective hydrolases released. Furthermore, the analysis of the secreted enzymes enabled identification of the motif with the consensus sequence LALL[LA]L[LA][LA]AAAAAAA as a potential signal peptide.

**Conclusions:**

Relative quantification of spectral intensities from crude proteomic datasets enabled the identification of new enzymes and provided new insights into protein secretion, as well as the molecular mechanisms of carbo-hydrolases involved in the cleavage of the selected carbon oligomers. These insights can help unlock new substrate sources for *C. oleaginosus*, such as low-cost by-products containing difficult to utilize carbohydrates. In addition, information regarding the carbo-hydrolytic potential of *C. oleaginosus* facilitates a more precise engineering approach when using targeted genetic approaches. This information could be used to find new and more cost-effective carbon sources for microbial lipid production by the oleaginous yeast *C. oleaginosus*.

**Supplementary Information:**

The online version contains supplementary material available at 10.1186/s12934-021-01692-2.

## Background

The oleaginous yeast *Cutaneotrichosporon oleaginosus* (*C. oleaginosus*, ATCC20509, *Agaricomycotina*) is currently the most promising microorganism for resource-efficient microbial lipid production, that can accumulate high contents of intracellular lipids (up to 87% w/ d.c.w.), using sustainable and inexpensive carbon sources [1]. The genus Cutaneotrichosporon belongs to the Basidiomycota family [2]. Usually, glucose is the preferred carbohydrate source of most microorganisms [3]. However, *C. oleaginosus* can metabolise various carbon sources in parallel (including pentoses) without catabolic repression [4]. In particular, *C. oleaginosus* features an increased capacity for metabolising diverse and chemically complex carbon sources, including most mono-, di-, and trimeric sugars, as well as glycerol, volatile fatty acids, and lignin-derived aromatics [4–6]. This unique attribute provides *C. oleaginosus* with the metabolic capacity to utilise complex biomass hydrolysates derived from various biomass sources, such as lignocellulose and crude glycerol derived from biodiesel processing, without the need for detoxification. Such biomass sources have been shown to contain well-established fermentation inhibitors, such as acetic acid, furfural, and methanol [1, 7, 8]. The wide acceptance and parallel use of different carbon sources enables the efficient metabolism of complex substrates obtained from biological waste streams, including macroalgal residues [9–11]. Interestingly, toxic substances have only a minor impact on the growth of this yeast, which indicates it employs rapid adaptation mechanisms. Thus, *C. oleaginosus* can tolerate and even metabolically utilise high concentrations of acetic acid, which is not the case for the model yeast *Saccharomyces cerevisiae* [1].

Currently, the molecular mechanisms enabling *C. oleaginosus* to metabolise an extremely broad range of carbon sources, including various oligosaccharides, are unknown. Commonly, yeast cells can absorb relatively small, monomeric carbohydrate molecules through specific membrane-associated transporters [12, 13]. Thus, high-molecular-weight carbohydrates (i.e., oligosaccharides and polysaccharides, such as maltose and cellulose) cannot be directly utilised, as they must initially be cleaved to yield fermentable monomeric glucose [14]. The utilisation of complex carbohydrates requires their extracellular degradation by hydrolytic enzyme components, which generate sugar monomers that can be imported into yeast cells and metabolically utilised [15]. Alternatively, filamentous fungi, such as *Trichoderma reesei*, are known to secret large amounts of hydrolytic enzymes into the surrounding environment, which enable the hydrolysis and subsequent metabolic utilisation of remote, chemically complex polymeric carbon sources [16, 17]. In other cases, cell-associated hydrolytic enzymes are found on the surface of bacterial microorganisms (such as *Clostridium thermocellum*) and organised as a cellulosome [14, 18]. Due to the short distance between the cell-associated hydrolytic complex and the substrate, the high concentrations of released monomers facilitate enhanced absorption by the host. In addition, the intracellular processing of these mobilised carbohydrates by specific enzymes is required for their metabolic utilisation. Although the metabolic routes for carbohydrate uptake and consumption have been described for model yeasts such as *S. cerevisiae* and *Pichia pastoris*, no equivalent data are available for *C. oleaginosus* [19–21].

Presently, the metabolism and processing of oligomeric carbohydrates are well described for many organisms [3, 22]; however, neither comprehensive nor systematic data are available for *C. oleaginosus*. The oligomeric sugars selected in this study, are common carbohydrates comprising glucose, galactose, and fructose. All sugarsare commercially available or can be released from the hydrolytic processing of lignocellulosic sources [23].

**Fig. 1:**
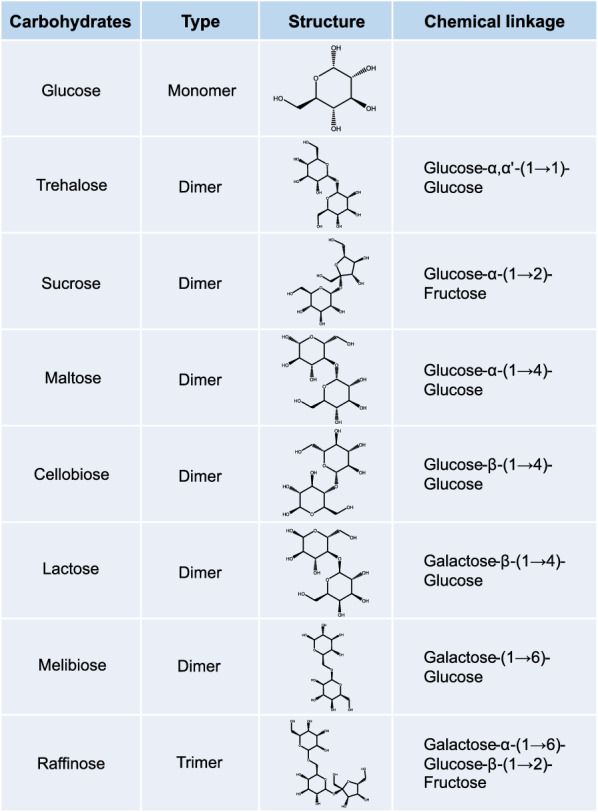
Overview of the carbon sources investigated in this study, their structures, and links providing further information on the type of connection as well as their monomeric structure

For example, cellobiose and sucrose can be obtained from hydrolysis of wood residues and molasses distillation, respectively [24, 25]. Maltose, in contrast, can be obtained from starch-containing products, such as potato, barley and cassava, while lactose is a constituent of milk whey [26, 27]. As a first step towards elucidating the biochemical and regulatory pathways underlying the metabolic utilisation of di- and trimeric carbohydrates in *C. oleaginosus,* a series of glucose epimers with different linkages (Fig. 1) were used as the sole carbon source for culturing *C. oleaginosus*. The data were complemented with a systematic morphological and proteome analysis. Initially, the *C. oleaginosus* growth rate and morphology were assessed based on the featured disaccharides. Subsequently, a comprehensive proteomic analysis of secreted membrane protein fractions, cell wall-associated protein fractions, and cytoplasmic protein fractions was carried out. A comparative analysis of the generated liquid chromatography–tandem mass spectrometry (LC–MS/MS) data revealed significant differences (p ≤ 0.05) between samples cultured in media containing the selected disaccharides, when compared to glucose controls. In addition, specific signal peptides for the secretion of hydrolytic proteins into the surrounding environment were identified, which represent potential targets for metabolic-engineering approaches.

## Results and discussion

### Evaluation of different carbohydrate effects on cellular growth, morphology and lipid formation as well as fatty acid distribution

The ability to assimilate extracellular carbohydrates is of crucial importance for most heterotrophic microorganisms. However, the cellular-uptake capacity and preference for certain carbohydrates depend on the natural habitat of the microorganism of interest and the prevailing environmental conditions. In complex carbohydrate mixtures such as biomass hydrolysates, the preferred carbohydrate is usually consumed first, whereas the metabolism of the other available carbohydrates is catabolically repressed [28]. Nevertheless, specific microorganisms, including *C. oleaginosus*, can explicitly metabolise different monomeric carbohydrates in parallel [28]. In contrast to the metabolic utilisation of monomeric carbohydrates, the use of oligomers as a carbon source has received little attention in the literature [4]. To gain insight into the metabolic mechanisms of carbohydrate oligomer utilisation, we conducted a comprehensive study to evaluate the growth kinetics, morphological changes, and enzyme-expression patterns of *C. oleaginosus* cells.

**Fig. 2:**
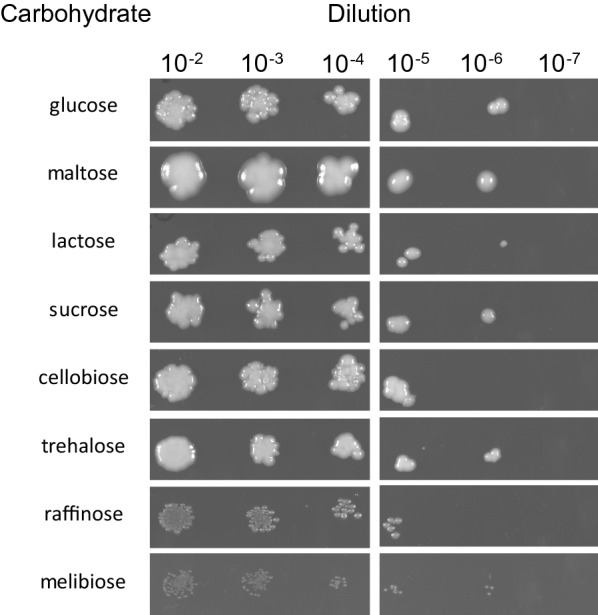
Analysis of* C.oleaginosus* growth using different carbohydrate substrates in the medium

Initially, *C. oleaginosus* cells were cultured on yeast nitrogen base (YNB) plates containing a set of different carbohydrates. The tested carbohydrates included glucose, maltose, lactose, sucrose, cellobiose, trehalose, raffinose, and melibiose. After two days, visible growth was observed for most carbohydrates at dilutions ranging between 10^–2^ and 10^–6^. Minimal growth was detected in plates containing melibiose and raffinose. Comparing the phylogeny of *C. oleaginosus* with typical lipid producing yeasts (Additional file 1: Fig. S1), *Trichosporon cutaneum* is the closest relative and in contrast to *C. oleaginosus* is able to metabolise melibiose in liquid medium [2, 29]. Furthermore, *C. oleaginosus* colonies grown on solid media containing maltose were significantly larger than those grown on media containing glucose, lactose, sucrose, cellobiose, or trehalose (Fig. 2).

**Fig. 3:**
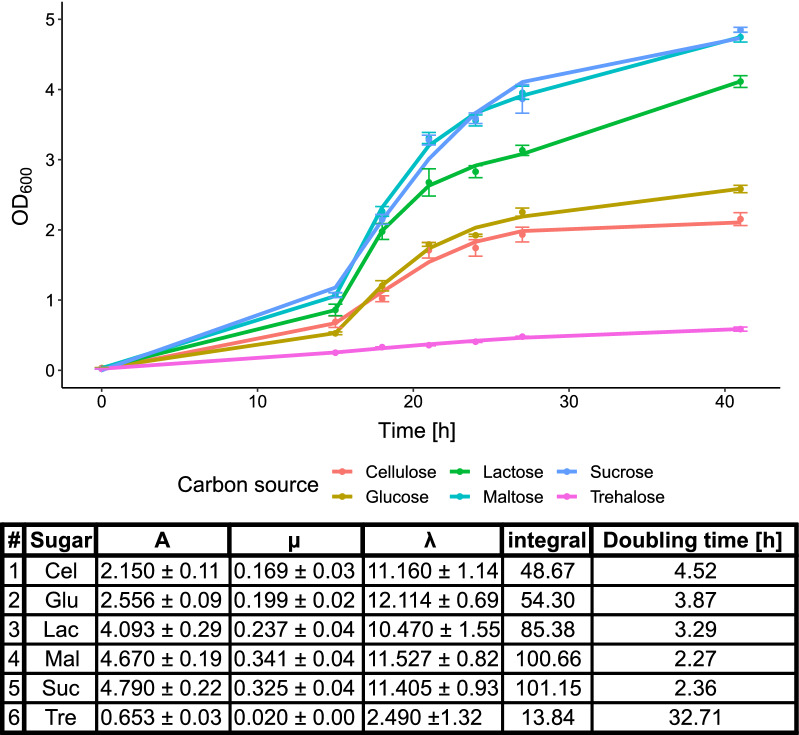
Upper panel: *C. oleaginosus* growth curves in media supplemented with different carbohydrates. Lower panel: max. growth (A), specific growth rate (µ), lag-phase (λ) and doubling time. The parameters were calculated in R using gompertz as model

As the colony size only provides limited information regarding the growth performance, a culture series was carried out in liquid media. The liquid cultures that used raffinose and melibiose as a carbohydrate substrate showed no growth. The growth as well as the specific growth rate, doubling time and lag-phase of *C. oleaginosus* cultures in the presence of the other disaccharides is depicted in Fig. 3. Differences in the growth patterns were visibly detected, depending on the carbohydrate substrate. *C. oleaginosus* cultures in liquid medium showed similar growth curves in the presence of sucrose and maltose. The sucrose and maltose samples reached final optical density at 600 nm (OD_600_) values of 4.75 and 4.85, which were twice as high as those cultured with glucose and cellobiose, respectively. Previously, other filamentous fungi such as *Mortierella isabellina* grew two times faster on glucose than on sucrose [30]. *C. oleaginosus* in media supplemented with sucrose, maltose, or lactose exhibited similar growth rates, with the latter showing the slowest growth. After 42 h, these cultures showed a final OD_600_ value of 4.11. Although cellobiose cultures grew similarly to the glucose reference cultures, a slightly reduced growth curve was observed with cultures supplemented with cellobiose, as confirmed by their final OD_600_ values of 2.15 and 2.58, respectively. These results differ from the growth-rate data for *T. cutaneum* described by Mörtberg and colleagues [29]. In contrast to *C. oleaginosus*, *T. cutaneum* grew almost identically in media supplemented with glucose, maltose, lactose, sucrose, cellobiose, or melibiose [29]. However, both strains showed similar decreases in their growth rates when the culture medium was supplemented with trehalose [29].

**Fig. 4:**
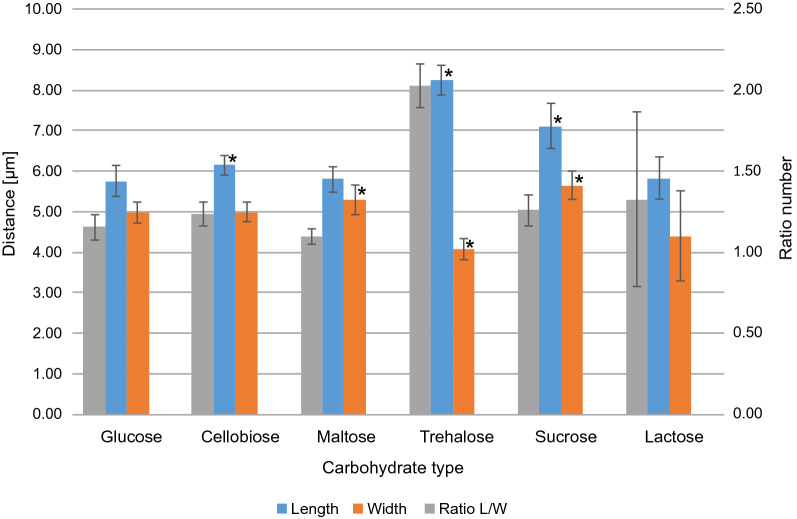
Morphological variations of *C. oleaginosus* in different culture media. The measured lengths and diameters of the *C. oleaginosus* cells in different cell media are shown. Additionally, the ratio length: width is shown on the secondary axis. The asterisks indicate a significant difference compared to the glucose condition (p < 0.05)

To examine morphological changes occurring with the yeast cells, the samples were collected during the exponential growth phase. Significant differences in length: width ratio were observed for cultures supplemented with cellobiose, maltose, sucrose, or trehalose compared to those supplemented with glucose (control; Fig. 4 and Additional file 2: Fig. S2). A significant increase in the cell length was observed in cellobiose-supplemented cultures, whereas the width increased in maltose-supplemented cultures. Furthermore, the cells cultured in media supplemented with sucrose or trehalose showed the biggest differences in the length: width ratio, indicating that an increase in the length occurred. Notably, the cultures supplemented with trehalose displayed a more round- to rod-shaped morphology, compared to that found in cultures supplemented with glucose. In the Basidiomycota, morphological changes are common in the course of sexual reproduction [31, 32]. Furthermore, changes in morphology of yeasts have already been demonstrated in *Yarrowia lipolytica* using N-glycosamine and serum to induce the formation of hyphaes [33, 34]. In addition, ambient temperature can affect cell size and morphology on *S. cerevisiae* [35]. Notably, this is the first study describing morphological changes in oleaginous yeasts in response to the supplied carbohydrate source during fermentation. In order to analyse the effects of different carbohydrates on lipid production, lipogenesis in *C. oleaginosus* was induced under nitrogen limiting growth conditions [4].The distribution of the identified fatty acids, as well as their quantities is shown in Additional file 3: Fig. S3a and b. The lowest fatty acid amount compared to the glucose control was detected in the cultures growing on trehalose with 9.5% (w/dcw, weight/dry cell weight). In contrast, lactose showed the highest fatty acid accumulation with 29.08% (w/dcw). Maltose, cellobiose and sucrose also resulted in elevated fatty acid yields compared to the glucose control (see Additional file 3: Fig. S3a). Furthermore, as described in a previous study, the fatty acid profile (Additional file 3: Fig. S3b) shows that maltose and lactose have a similar fatty acid distribution as the glucose control [4]. However, a slight reduction in oleic acid, as well as an increase in palmitic acid and stearic acid, as described by Awad, could be detected [4]. The most significant influence on the fatty acid profiles could be observed in cultures containing Trehalose. Interestingly, for the Trehalose grown cultures a shift in the proportion of linoleic acid and α-linoleic acid by a factor of 1.58 and 3.12 respectively could be detected with reference to the glucose control. Moreover, the proportion of oleic acid was reduced by a factor of 0.72 compared to the reference culture. At present, there is no biochemical data that could be used to delineate this effect. Currently, detailed metabolomics studies are underway in our research group to address the required data sets.

### Effects of different disaccharides on the distributions of enzyme classes

The effects of different disaccharides on selected protein fractions isolated from the cytoplasm, cell wall, and secretions of *C. oleaginosus* were analysed. The selected carbon sources directly influenced the types and quantities of proteins expressed in *C. oleaginosus*, thus providing information regarding the uptake and metabolism of the respective carbon source.

The high data quantity and quality generated in this study enabled sequence-based analysis of the identified proteins. Initially, overlapping and fraction-specific proteins for the selected carbohydrates were analysed and visualised using Venn diagrams (Additional file 4: Fig. S4). In the secreted fraction of cells cultured in medium supplemented with cellobiose or lactose, the highest numbers of total and carbohydrate-modified proteins were detected. Additionally, an ontology-based classification of the proteins into classes such as biological processes, molecular functions, and cellular components was performed (Additional file 5: Fig. S5. Additional file 6: Fig. S6, Additional file 7: Fig. S7). Within this dataset, an approximately equal distribution of each protein group was observed, whereas the total number of proteins per group varied.

**Fig. 5:**
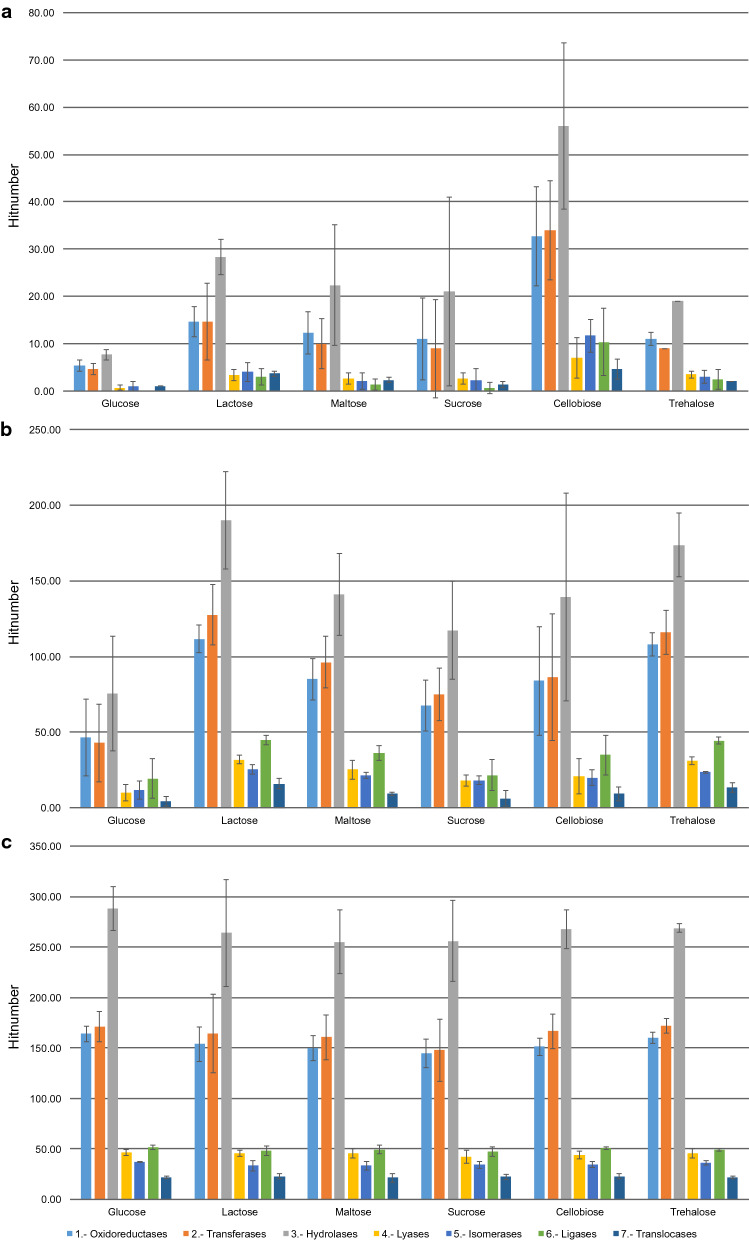
Enzyme hit distribution. For each carbohydrate, the numbers of different enzymes identified (based on the EC number) are shown for the secreted, cell wall-associated, and cytoplasmic fractions

To functionally analyse the identified proteins expressed under specific carbohydrate conditions and compare them to the reference condition (glucose), a classification of their respective enzyme commission (EC) numbers was performed. The subsequent assignments of each fraction (secreted, cell wall-associated, and cytoplasmic), as well as the respective carbon source, are shown in Fig. 5. The EC numbers did not vary significantly for the cytoplasmic fraction when compared to those of the reference condition (Fig. 5c); however, the EC number distributions of the cell wall-associated enzyme fractions were significantly different when comparing the diverse carbohydrate sources with that of the reference condition (Fig. 5b). The lactose and trehalose datasets indicated a two-fold enhancement in various hydrolase activities (enzyme class 3). Significant differences in the EC numbers were also detected in yeast cultures in medium supplemented with maltose, except for the class of ligases (enzyme class 6).

Interestingly, the distributions of the enzyme classes found in the secretome showed the greatest quantitative differences. This contrasts the situation in the yeast *P. pastoris*, whose secretion profile hardly changed, when cultivated with different carbon sources [36]. As shown in Fig. 5a, a relatively small number of proteins per enzyme class could be detected under the glucose condition (≤ 10 different enzymes per enzyme class). Compared to the glucose condition, significant increases in the diversity of different enzyme classes were detected with all disaccharides investigated. Significant increases in enzyme diversities were observed under all conditions, particularly with enzyme class 1 (oxidoreductases) and class 4 (lyases). Notably, lactose and cellobiose showed significant differences in their hit numbers within all enzyme classes; lactose and cellobiose showed a four-fold increase in the number of enzyme class 3 (hydrolases) and a seven-fold increase in lactose and cellobiose, when compared to those observed under the glucose condition.

For an in-depth analysis of the hydrolases used to cleave individual disaccharides, the enzymes of subclass EC 3.2 (glycosylases) were further investigated.

### Influences of distinct carbohydrates on the expression of secreted hydrolases

**Fig. 6:**
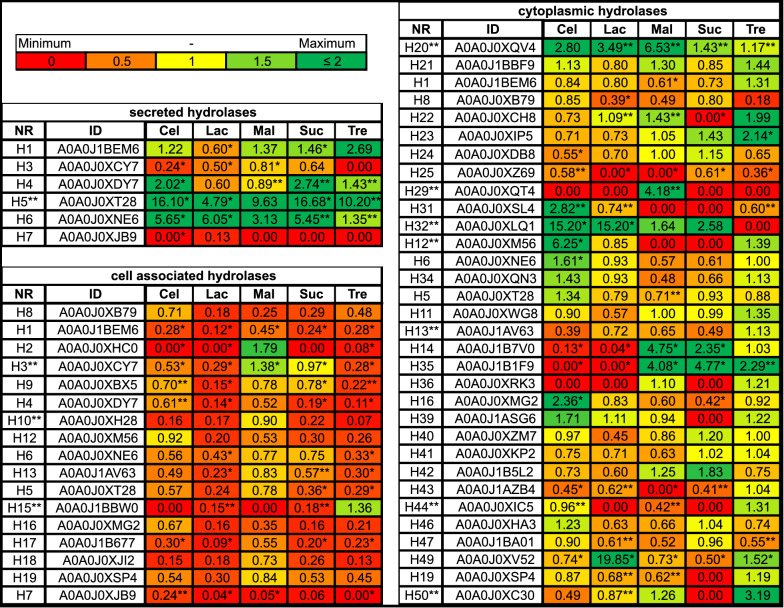
Relative expression levels of the hydrolases identified under each carbohydrate condition, normalised to the expression levels measured under the glucose condition. The identified putative hydrolases are listed, and their relative expression levels compared to that observed under the glucose condition are indicated. Significant differences (p < 0.05) are indicated with an asterisk. Values based on duplicates are marked with two asterisks

**Fig. 7:**
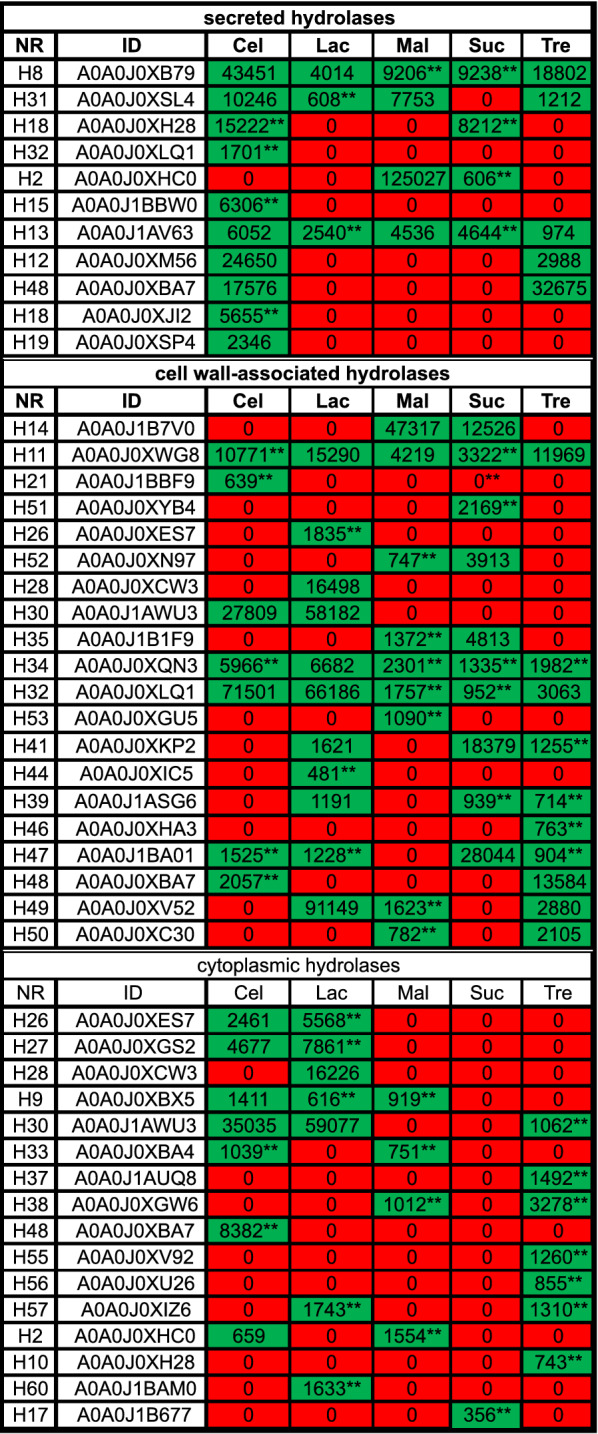
Expression levels of the identified hydrolases. The hydrolases detected exclusively in the disaccharide-supplemented cultures are shown

A detailed proteomic analysis of the effects of sugar substrates on the expression of hydrolytic enzymes revealed significant differences, when comparing the glucose condition with the other carbohydrate conditions. Figure 6 shows that the hydrolases identified under the glucose condition were downregulated compared to those observed in cultures grown in disaccharide-containing media. However, as shown in Fig. 7, numerous enzymes were specifically produced that could potentially cleave the investigated disaccharides. The predictions of the potential functions of the identified enzymes are shown in Additional file 8: Fig. S8.

#### Cellobiose

For the secretome cultured in media supplemented with cellobiose, a significant upregulation of the proteins H4 (ß-1,3–1,4-glucanase), H6 (glucan endo-1,3-ß-glucosidase), and H5 (α-amylase) was observed. Six-fold and two-fold upregulations in H6 and H4 were observed, and these proteins shared 29.4% and 44.29% sequence identity with previously reported ß-glucosidases, respectively [37, 38]. Moreover, H5 was the most upregulated (16-fold) under the cellobiose condition (when compared to the glucose condition), and it shared 39.32% sequence identity with α-amylase [39]. In addition, the hydrolase H15 (exo-ß-(1,3)-glucanase), which was only detected in cultures grown in cellobiose-containing media, exhibited a 42.5% sequence identity to a ß-bond-cleaving enzyme [40].

#### Lactose

Secretome analysis of the cells cultured in lactose-containing media also showed upregulation of the H6 and H5 proteins. The four-fold upregulation of hydrolase H5 was lower than that observed in cells cultured in cellobiose-containing media; however, it differed significantly from the expression observed in cells cultured in glucose-containing media. These results indicate that the ß-glucosidase H6 was released by *C. oleaginosus* cells for the cleavage of different disaccharides linked by β-1,4 glycosidic bonds. These findings are consistent with the results of Nakkharat et al., who demonstrated that ß-glucosidase from the thermophilic fungus *Talaromyces thermophilus CBS 236.58* can also exhibit ß-galactosidase activity [41].

#### Maltose

Using maltose as the carbon source led to upregulation of the H5 and H6 proteins in the secreted protein fraction of *C. oleaginosus* cultures. H5 expression was upregulated nine-fold compared to that observed under the glucose condition. Furthermore, in silico analysis identified H5 as an α-amylase responsible for maltose cleavage. In addition, the H2 enzyme (α/ß-glucosidase) was detectable almost exclusively in *C. oleaginosus* cultured in maltose-supplemented medium. This enzyme might cleave the α position of sugar compounds due to its 39.3% sequence identity match with a previously described α/ß-glucosidase [37].

#### Sucrose

Analysis of the secreted protein fraction of cultures grown in sucrose-containing media revealed upregulation of proteins H1 (endo-1,3(4)-ß-glucanase), H4, H5, and H6. H5 and H6 were the most upregulated proteins, exhibiting 17-fold and five-fold increases in expression, respectively. Surprisingly, although it cleaves sucrose, the H3 protein (invertase), which partly corresponds to an invertase, was downregulated. Since *C. oleaginosus* cells showed efficient growth in sucrose medium, this finding suggests that sucrose might not be hydrolysed extracellularly by *C. oleaginosus* cells; instead, membrane-associated, or intracellular processes might perform this function. This is in line with previously published data [29] describing the incapacity of *T. cutaneum* to hydrolyse sucrose extracellularly.

#### Trehalose

*C. oleaginosus* cultured in media supplemented with the disaccharide trehalose showed upregulation of the H1 (endo-1,3(4)-beta-glucanase), H4, H5, and H6 proteins in the secreted fractions. Although a relatively high upregulation of the H1 and H5 proteins was detected (17-fold and seven-fold, respectively), these proteins do not seem to be involved in trehalose hydrolysis, as indicated by the negative enzyme test.

The results of the substrate tests with concentrated protein fractions from the *C. oleaginosus* cultures are shown in Additional file 9: Fig. S9. The enzymatic tests indicated that *C. oleaginosus* can extracellularly hydrolyse disaccharides such as cellobiose, lactose, and maltose. However, sucrose and trehalose do not appear to be hydrolysed by the secreted proteins in the respective cultures. These results distinguish *C. oleaginosus* from the closely related yeast *T. cutaneum*, which cannot extracellularly hydrolyse cellobiose, lactose, and maltose, but can hydrolyse sucrose and trehalose [29].

### Influences of different carbohydrate sources on membrane- and cell wall-associated hydrolases

Membrane- and cell wall-associated proteins play key roles in the recognition and uptake of extracellular carbohydrates [42]. A proteome analysis was performed to further elucidate the protein abundances and expression patterns in *C. oleaginosus*.

#### Cellobiose

All hydrolases detected in *C. oleaginosus* cultured in glucose-containing media were downregulated when cultured in media supplemented with cellobiose. However, as shown in Fig. 7, the H11 (1,4-alpha-glucan-branching enzyme), H21 (α/ß-hydrolase), H30 (ß-glucosidase), H34 (glycoside hydrolase), H32 (ß-mannosidase), H47 (α-amylase), and H48 (ß-galactosidase) enzymes (which were not detectable under the glucose condition) were identified. The presence of the H30, H32, and H47 enzymes is of importance because they might be able to cleave the ß-bound cellobiose, based on reference database entries [43–45].

#### Lactose

The H26 (α-galactosidase), H28 (ß-galactosidase), H30, H34, H32, and H47 enzymes were identified under the lactose condition, but not under the reference glucose condition. H26 and H28 shared over 45% sequence identity to galactosidases detected in *Paecilomyces thermophila* and *Arthrobacter sp.* respectively [37, 46]. Furthermore, under the lactose condition, the hydrolase H30 was upregulated three-fold compared to that observed under the cellobiose condition. Like hydrolase H6, this hydrolase seems to be able to cleave cellobiose and lactose.

#### Maltose

The H2 (α/ß-glucosidase) and H3 enzymes were upregulated in yeast cultured in maltose-containing media, compared to yeast cultured in glucose-containing media. The changes in responses to maltose were most prominent amongst the responses to the other disaccharides. The H2 enzyme, which appears to play a role in the extracellular hydrolysis of maltose, was upregulated two-fold. In addition, enzymes such as H14 (α-glucosidase), H52 (glycosyl hydrolase), H35 (α-glucosidase), or H34 were over 36% identical to putative maltose-cleaving enzymes (Fig. 7). In particular, H14 displayed a six-fold upregulation and shares homology to the α-glucosidase expressed in *Halomonas sp.* [47].

#### Sucrose

The H35, H51 (α/ß-hydrolase), H52, H47, and H14 enzymes play dominant roles in the hydrolysis of sucrose, a carbohydrate with high relevance to industrial fermentation. The H47 enzyme-expression level was at least 28-fold higher under the sucrose condition than under other culture conditions. Furthermore, based on its homology to α-glucosidase, H14 might be able to cleave maltose and sucrose [47]. We could further infer that other enzymes, such as H52 and H35, are also formed in the presence of sucrose and maltose (Fig. 7). This may indicate that both disaccharides partly induce the same protein-expression profiles in *C. oleaginosus*. This bifunctionality has already been described by Williamson et al. for other yeast maltases [48]. In this study, a potential α-cleaving enzyme, H51, was exclusively detected under the sucrose condition.

#### Trehalose

When the culture medium was supplemented with trehalose, the H11, H32, H48, H50 (neutral trehalase), and H46 (α-glucosidase) enzymes were strongly expressed. Notably, the H46 enzyme was only detected under the trehalose condition. Furthermore, this enzyme exhibits 45% sequence identity to a previously described α-glucosidase [49]. Moreover, the H50 enzyme exhibits a 50% sequence identity to the yeast trehalase identified by Alblova et al. [50]; its expression was upregulated at least three-fold in *C. oleaginosus* cultured in trehalose-containing media compared to that observed in *C. oleaginosus* cultured in glucose-containing media.

### Influences of different carbohydrates on the expression of soluble cytoplasmic hydrolases

To gain a better understanding of intracellular carbon utilisation, cytoplasmic enzymes were further examined. The obtained data are presented in Figs. 6 and 7, and the regulations and annotations of the identified cytoplasmic enzymes are illustrated.

#### Cellobiose

A detailed examination of the cultures obtained from cellobiose-containing media indicates that nine potential hydrolases are upregulated. The H20 (α/ß-hydrolase), H31 (glucan 1,3-beta-glucosidase), H32, and H12 (exo-ß-(1,3)-glucanase) enzymes showed at least two-fold higher expression levels compared to those observed in cultures obtained from glucose-containing media. The corresponding identities match at least 34% with ß-cleaving enzymes. The most dramatic change was observed for the H32 enzyme, which was identified in the other fractions. This specific putative ß-mannosidase, which matches a ß-mannosidase detected in *Myceliophthora thermophila* [44], showed a 15-fold upregulation in cultures obtained from cellobiose-containing media. Moreover, H30 was also detected in the cytoplasmic fraction when the yeast cells were cultured in media supplemented with cellobiose or lactose.

#### Lactose

A similar expression pattern can be induced by lactose in the culture medium. In lactose-containing media, the H32 enzyme was the most upregulated, and the H30 enzyme-expression level increased 1.4-fold compared to that observed in cultures obtained from cellobiose-containing media. The formation of H32 and H30 in cellobiose- and lactose-based cultures indicates that *C. oleaginosus* utilises these enzymes to cleave cellobiose and lactose, respectively. Furthermore, it can be concluded that *C. oleaginosus* expresses specific enzymes to cleave various ß-bound monosaccharides. Interestingly, H28 was only detected in cultures grown in lactose-supplemented media. Finally, H28 shares 45% sequence identity with a ß-galactosidase previously detected in *Arthrobacter sp. 32cB* [46].

#### Maltose

Under the maltose condition, the expression levels of the H20, H14, and H35 enzymes were upregulated two-fold compared to those observed under the glucose condition. These enzyme sequences correspond to α-cleaving enzymes, which might play a role in cytoplasmic maltose cleavage. Further, H14 and H35 match the sequences of well-characterised glucosidases identified in the *Halomonas* sp. and *Bacillus cereus* bacteria [47, 51]. In addition, the data in Fig. 7 indicate the formation of the H2 enzyme, which might play a dominant role in the previously discussed secretomes of maltose-based yeast cultures.

#### Sucrose

As shown in Fig. 6, sucrose induced the expression of the H14, H35, H42 (acylpyruvate hydrolase), and H32 enzymes. The H14 and H35 enzymes showed at least 36% sequence identity to the α-glycosidases previously identified in *Halomonas sp.* and *Schizosaccharomyces pombe* [47, 52]. In addition, H20 was identified as an enzyme with 59.5% similarity to the sequence of a modelled a/β hydrolase.

#### Trehalose

Finally, the formation of α-1,1-bound trehalose-cleaving hydrolases was analysed. The H22 (putative dienelactone hydrolase), H23 (α/ß-hydrolase), and H35 (α-glucosidase) enzymes were upregulated two-fold under the trehalose condition, compared to the glucose condition. The H23 enzyme shares 51% sequence identity with an α/β hydrolase. Furthermore, as shown in Fig. 7, the H55 (α/ß-hydrolase), H56 (α/ß-hydrolase), and H10 (glucan endo-1,3- α-glucosidase agn1) enzymes were only detected in the cytoplasmic fraction of *C. oleaginosus* cultured in trehalose-containing media. H55 and H56 share over 50% homology with α/β-hydrolases. These enzymes can cleave the α-1,1 bond of trehalose into glucose monomers. In addition, the expression of the potential trehalase H50, which was identified in the cell wall-associated fraction, was increased three-fold under the trehalose condition, compared to that under the glucose condition. Enzymes such as H2, H30, and H26 were disregarded from the analysis since they can cleave other disaccharides. Although the H2 enzyme was produced in the secretome of cultures obtained from trehalose-containing media, the dinitrosalicylic acid (DNS) test did not detect H2-specific activity.

### The *C. oleaginosus* hydrolase composition enabled metabolic utilisation of chemically distinct disaccharides

**Fig. 8:**
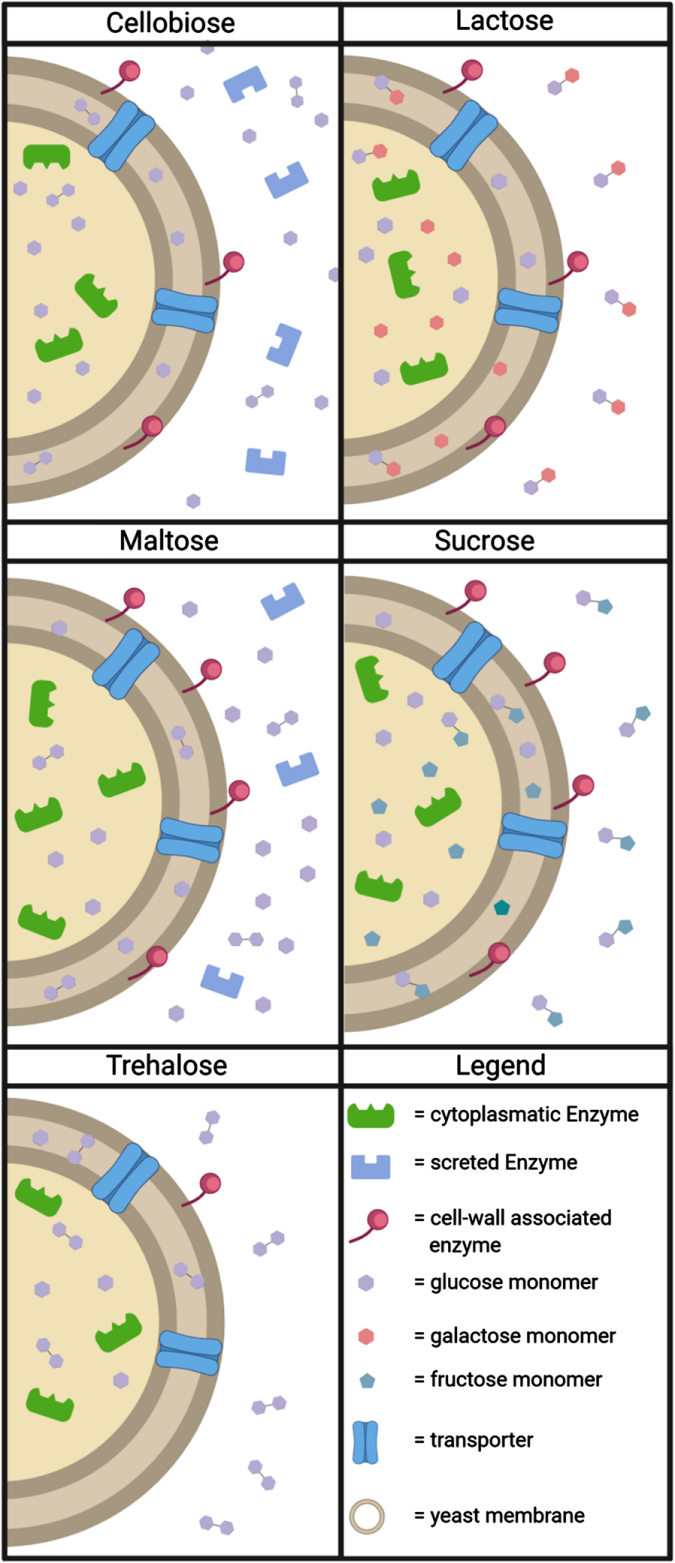
Representative model of disaccharide cleavage and uptake in *C. oleaginosus*. The model illustrates the potential cleavage in the subcellular or extracellular compartment and subsequent uptake of carbohydrates by *C. oleaginosus*. The model schematically indicates which fractions (secreted, cell wall-associated, or cytoplasmic) contained the enzymes necessary for cleaving the indicated disaccharides

Conducting a detailed analysis of the carbohydrate metabolism is the first step in understanding the versatile substrate use of *C. oleaginosus*. Therefore, we investigated different dimers and trimers, among the glucose epimers. In the following sections, the obtained data are interpreted, and the results are graphically summarised in Fig. 8.

#### Cellobiose

Yeast cultures grown in cellobiose-based medium reached an OD_600_ of 2.15 after 42 h, which was the second-lowest value observed among all carbohydrate-based medium conditions tested. All analysed fractions contained different putative cellobiose-cleaving enzymes. The reduced growth on liquid cellobiose-containing medium could be explained by the higher galactosidase activity of the identified ß-1–4 cleaving enzymes. This finding further explains the expression of these hydrolases in *C. oleaginosus* cells cultured in lactose-containing medium. Furthermore, it has been documented that the same glycosyl hydrolase activity can cleave several carbohydrates with different substrate affinities [41]. In addition, the high number of ß-glucosidases expressed suggests that *C. oleaginosus* might cleave cellobiose intracellularly. This possibility is further supported by observations that cellulolytic fungi can carry out intracellular hydrolysis of cellobiose [53].

#### Lactose

Yeasts cultured in lactose-supplemented media reached an OD_600_ of 4.11, which was two-fold higher than that observed under the cellobiose condition. Since many enzymes are expressed during growth based on either carbohydrate, we hypothesise that they have a higher enzymatic activity for the substrate lactose. In addition, *C. oleaginosus* exhibited increased expression of enzymes such as H30 and H20 in lactose-based cultures, when compared to that in cellobiose-based cultures. An indication of the localisation of lactose cleavage in *C. oleaginosus* is provided by the observation that galactosidases (such as H26 and H28) were produced, which are mainly found in the membrane and in the cytoplasm. In addition, an associated lactose permease was identified, which indicates that *C. oleaginosus* might primarily degrade lactose at the membrane or intracellularly. However, Carvalho-Silva and Spencer-Martins showed that lactose can be cleaved extracellularly in various *Kluyveromyces marxianus* strains [54]. Positive activity for lactose cleavage in the secreted fractions of *C. oleaginosus* (Additional file 9: Fig. S9) further supports this observation. In contrast, the intracellular utilisation of this carbohydrate differs in *C. oleaginosus* from that in the related strain *T. cutaneum*, which predominantly degrades lactose extracellularly [29].

#### Maltose

For yeast cultured in maltose-containing medium, the highest biomass increase was determined at an OD_600_ of 4.75, whereas sucrose-based cultures reached an OD_600_ of 4.85. The significant increase in H2 expression in the presence of maltose in the secretome highlights the efficient cleavage of this disaccharide. Additional enzymes, such as H20 and H14, were upregulated in the membrane and cytoplasm. These findings support the conclusion that *C. oleaginosus*, similar to *Cryptococcus flavus*, secretes amylases and efficiently cleaves maltose extracellularly [55]. However, this is not the case for *T. cutaneum* and many brewery yeasts [29, 56]. Since *C. oleaginosus* enzymes such as H20 and H14 are upregulated in the membrane and cytoplasm, other metabolic pathways are activated in parallel to the extracellular cleavage of maltose [57].

#### Sucrose

Compared to other disaccharide-based cultures, the cultures grown in sucrose-based medium showed the highest cell density, with an OD_600_ of 4.85 after 42 h. Furthermore, our current analysis indicated that *C. oleaginosus* cannot degrade sucrose extracellularly, in contrast to *S. cerevisiae* [58]. However, some potential sucrose-cleaving enzymes, such as H11, H14, H20, and H35, were identified in the membrane and cytoplasmic fractions. These findings suggest that *C. oleaginosus* cleaves sucrose substrate intracellularly and/or via a membrane-associated process. This hypothesis is supported by positive enzyme activity observed in the cell wall fraction, as well as numerous studies that have investigated the utilisation of sucrose in yeast [58].

#### Trehalose

Among the disaccharides investigated, the trehalose-based cultures showed the slowest growth. Although a high number of different hydrolases were observed in the membrane and cytoplasmic fractions of *C. oleaginosus*, only the detected trehalases are related to trehalose cleavage and cell growth. In addition, trehalose is often used as a storage compound or formed as part of a stress response towards dehydration or increased ethanol levels [59]. This suggests that trehalose is less suitable for *C. oleaginosus* cultivation.

### Analysis of secreted proteins

Proteins targeted toward the secretory pathway are mediated by the endoplasmic reticulum (ER)–Golgi endomembrane pathway. This is the first step in the recognition of signal sequences (such as the signal-recognition particle) and association with the ER [60]. Further protein sorting within the endomembrane system is regulated by specific sequence motifs, followed by specific glycosylation processes [61]. Thus, identifying these sequence motifs is important for improving the understanding of subcellular protein sorting and subsequent protein secretion.

To identify new signal motifs, all 1,091 secreted proteins with a -10 · lgP value higher than 36 were analysed for potential signal peptides using the SignalP5.0, Phobius, and WoLF PSORT software programs. These analyses identified 112 proteins with potential eukaryotic signal-peptide motifs. These hits represented candidate signal sequences that potentially function to introduce proteins to the secretion pathway by triggering their transport into the ER.

**Fig. 9:**
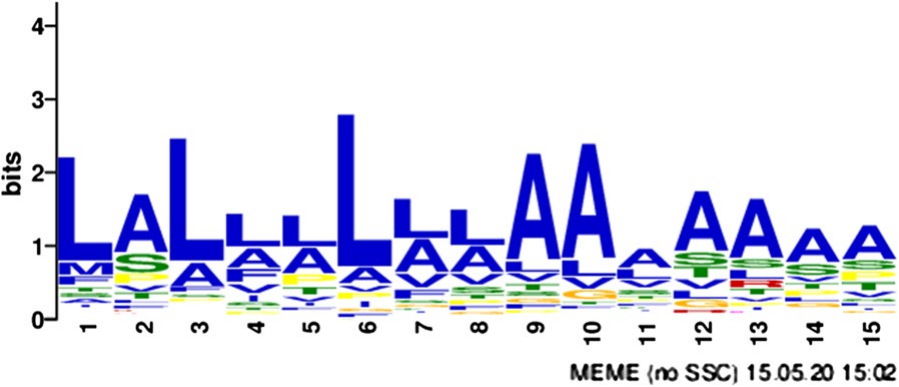
Identification of the LALA motif (Logo). The LALA motif was identified in the N-terminal sequences of 81 of 112 potential signal peptide sequences, using MEME Suite 5.1.1 [88]

The 112 hits were further analysed using the MEME tool (from MEME Suite 5.1.1) for novel motif elicitation against a background dataset of the 1,000 most abundant cytoplasmic enzymes. A 15 amino acid-long motif was identified, which was present in 81 of the 112 proteins (p = 9.4e-007). The motif comprises a stretch of the hydrophobic amino acids, alanine and leucine, with the consensus sequence LALL[LA]L[LA][LA]AAAAAAA (Fig. 9). Furthermore, the motif is primarily located at the amino termini of the proteins, as a part of the predicted signal peptide, which have a hydrophobic character [62]. Nevertheless, in eight cases, the motif was also found at the C-terminal side and in some rare cases, in the middle of the protein.

Amongst the 1,091 secreted proteins selected, the consensus motif was found in 358 cases (p < 0.0001). When screening for the motif within the 4,498 cytoplasmic proteins detected in this study, 1,107 hits were obtained. These findings might suggest that the motif belongs to a signal peptide that triggers transport into the ER, but has no direct influence on protein secretion itself. To trigger protein secretion from the ER to Golgi and out of the cell, another signal is required, such as a pro-peptide with a certain glycosylation pattern. For the yeast *S. cerevisiae,* a combination of signal peptides (pre-peptide and pro-peptide) similar to the glycosylated pro-α-mating factor can lead to protein secretion [63]. Moreover, *P. pastoris* is an established host for the production of recombinant, secreted proteins [64]. The signal sequence for secretion is reported and can be fused to the recombinant protein to initiate cellular export. In addition, *Y. lipolytica* is also able to efficiently secrete proteins. Several secretion signals have been described and corresponding expression systems generated [65–68].

In the secreted protein fraction, glycosylated peptides could be identified (Additional file 12: Table S1). Within this dataset, the most abundant carbohydrate-based post-translational modifications were derived from glucose (delta molecular weight [MW]: 162.05), heptose (delta MW: 192,06), hexosamine (delta MW: 161.07), gluconoyl (delta MW: 178.05), and fucose (delta MW: 146.06). Within this dataset, numerous carbohydrate-related proteins that are potentially involved in cellular export were discovered. In addition, some of these glycolytic motifs were identified in hydrolases H1, H6, H8, H13, H12, H31, and H32.

## Conclusions

The presence, uptake, and utilisation of different disaccharides in *C. oleaginosus* were studied using a comprehensive approach involving proteomic and morphological analyses. In this work, we addressed the influence of a selected set of different di- and trisaccharides as carbon sources for the first time in *C. oleaginosus*, followed by a wide proteomic analysis of expressed proteins. In addition, the target compartments of the proteins were considered, including cytoplasmic, cell-associated, and secreted proteins. Based on spectral information, the relative quantification of specific proteins was possible, which provided insights into the potential functions of the newly identified proteins. The subsequent assignment of the individual proteins to their enzyme classes helped uncover information regarding their formation and functions. Significant differences in the relative abundances of each substrate in the different fractions were also detected. For example, the addition of lactose or cellobiose—compared to the glucose reference—respectively resulted in three- and five-fold higher diversities of hydrolases (enzyme class 3) in the secretome. Significant increases in expression, such as the formation of H2 (α/ß-glucosidase) when maltose was added or H20 (α/β-hydrolase) and H30 (β-glucosidase) when cellobiose and lactose were used as substrates, were observed. In conclusion, the most prominent enzymes including H26, H28, H30, H32 and H47, exhibiting α-galactosidase, β-galactosidase, β-glucosidase, β-mannosidase and α-amylase activities respectively are summarized in Additional file 10: Fig. S10. Furthermore, a phylogenetic comparison of the identified hydrolases has revealed sequence and functional relationships (Additional file 11: S11). For example, the annotated α/β-glucosidase activity is found in the nearby branch of H2, H20, H21 and H25. Moreover, the annotated β-galactosidase activity is associated with the nearby branch of H28 and H30. The cumulative phylogenetic comparison therefore increased the confidence in our functional assignment of the identified proteins. In addition, by analysing the secreted enzymes, a motif with the consensus sequence LALL[LA]L[LA][LA]AAAAAAA was identified as a potential signal peptide. These insights reveal potential new substrate sources for *C. oleaginosus*, such as inexpensive metabolic by-products containing utilizable carbohydrates. Gathering information regarding the carbo-hydrolytic potential of *C. oleaginosus* allows for more precise engineering approaches. This information could be used to discover more cost-effective carbon sources for microbial lipid production by *C. oleaginosus*.

## Methods

### Spot assay for *C. oleaginosus*

A liquid culture of *C. oleaginosus* containing 10^7^ yeast cells/mL was diluted from 10^–2^ to 10^–10^ and spotted on solid media plates containing 6.7 g/L YNB (Carl Roth GmbH + Co. KG, Karlsruhe, Germany), supplemented with 20 g/L of the indicated carbohydrates and 20 g/L agarose. The plates were incubated for two days at 28 °C.

### *C. oleaginosus* cultures

*C. oleaginosus* (ATCC 20,509) cells were cultured in media containing YNB (6.7 g/L) supplemented with 20 g/L of the indicated carbohydrates. The yeast were cultured in baffled Erlenmeyer flasks on a rotary shaker at 120 rotations/min and 28 °C. The cells were inoculated in 30 mL culture medium containing glucose and incubated for 24 h. The pre-cultures were used to inoculate 500 mL baffled Erlenmeyer flasks containing 150 mL YNB medium at an OD_600_ of 0.1, which were then incubated for 3 days. The cells were harvested via centrifugation at 6,738 × *g* for 7 min, re-suspended in 150 mL fresh medium (without carbohydrates), and transferred in baffled Erlenmeyer flasks. After the addition of maltose, lactose, cellobiose, sucrose, trehalose, or glucose (control), the cells were cultured for 2 additional days. Each experiment was carried out in triplicate. The cultivation for the fatty acid analysis was carried out under the same conditions with an elongated cultivation time of 5 days under limiting conditions by reducing the ammonium sulphate content (nitrogen limitation) from 5.0 to 0.613 g/l.

### Fatty acid analysis

The fatty acid analysis was carried out according to the method of Griffiths et. al. with the modifications of Woortman et. al. [69, 70].

### Nile red staining and microscopy

The yeast cells were photographed using a microscope (Axio Lab.A1, Carl Zeiss AG, Oberkochen, Germany). Nile red staining was performed according to a previously described method [71].

### Protein extraction

Initially, secreted proteins were separated from the cells by centrifugation at 6,738 × *g* for 7 min. Cell-associated proteins were washed off the cells by re-suspension in 25 mL NaCl (150 mM). After a 10 min incubation at room temperature (RT, approximately 22 °C), the associated protein fraction was separated from the cells by centrifugation at 20,133 × *g* for 7 min. The remaining cell pellet was re-suspended in 15 mL NaCl (150 mM) and disrupted using a high-pressure homogeniser (8 bar; Avestin Europe GmbH, Mannheim, Germany). The soluble cytoplasmic protein fraction was separated from the cell debris by centrifugation at 20,133 × *g* for 7 min. In the second step, the secreted, membrane-associated, and cytoplasmic protein fractions were each filtered through a 0.45 μm filter to remove residual cellular material, precipitated with 10% trichloroacetic (v/v) acid for 30 min at 4 °C, and subsequently centrifuged at 20,133 × *g* for 10 min at 4 °C. The precipitated proteins were washed twice with 10 mL methanol (high-performance liquid chromatography [HPLC] grade) and three times with 10 mL acetone (HPLC grade). In each washing step, the precipitated proteins were centrifuged at 20,133 × *g* for 10 min at 4 °C. Finally, each pellet was dried at RT and dissolved in 30 μL urea (8 M). The protein concentrations were determined by measuring the 260: 280 nm absorbance ratios (NanoPhotometer NP80, Implen GmbH, München, Germany).

### Preparing native proteins from secreted fractions

Proteins were separated from secreted and membrane-associated fractions. Subsequently, the secreted and membrane-associated protein fractions were filtered through a 0.45 μm filter to remove residual cellular material. Further, 2.5 mL of each fraction was subjected to a buffer exchange in 25 mM 3-(N-morpholino) propane sulfonic acid (MOPS), with 50 mM NaCl (pH 6.2) for the secreted fraction and 25 mM MOPS with 150 mM NaCl for the membrane-associated protein fraction (pH of 6.2), using PD-10 desalting columns (GE Healthcare, Chicago, IL, USA). Each fraction was then eluted in a volume of 3.5 mL. After repeating the last step three times, 14 mL of each eluted protein fractions was concentrated to a final volume of 1.5 mL using a Centriprep 10 k filter (Merck Millipore, Darmstadt, Germany). All steps were carried out at 4 °C.

### DNS assay

The hydrolytic activities in the generated enzyme mixtures were determined using a DNS assay [72]. Ten microliters of the DNS reagent (300 g/L sodium potassium tartrate and 10 g/L 3,5-DNS in 0.4 M NaOH) was added to 30 µL of each sample and incubated for 10 min at 95 °C in a thermocycler. The released reducing sugars were measured spectrophotometrically at 540 nm. Briefly, the reactions were conducted in a volume of 500 µL, comprising the enzyme mix and 5 mg/mL of the tested sugars. Each sample was incubated for 72 h at 28 °C.

### Preparing protein samples for LC–MS/MS analysis

Proteins were run 1 cm into a 10% Criterion™ Tris–HCl Protein Gel (Bio-Rad Laboratories, Inc., Hercules, CA, USA) and subsequently stained with Coomassie Brilliant Blue (SERVA Electrophoresis GmbH, Heidelberg, Germany). Visualised protein bands were excised from the gel and used for peptide isolation according to a previously described method [73, 74], with modifications. The excised gel was shredded into small pieces (< 1 mm^3^) and washed with acetonitrile until the Coomassie Brilliant Blue was completely removed. Subsequently, the gel pieces were dried under vacuum for 15 min (GeneVac Evaporator, GeneVac HiTechTrader, Ipswich, United Kingdom). Samples were reduced (10 mM dithiothreitol and 50 mM ammonium bicarbonate) at 56 °C for 30 min, washed with acetonitrile, and alkylated (55 mM iodoacetamide and 50 mM ammonium bicarbonate) at RT for 20 min. After a second washing step with acetonitrile, the samples were dried under a vacuum for 15 min (SpeedVac) and rehydrated in digest solution containing Trypsin Gold (V5280, Promega, Madison, WI, USA), according to the manufacturer’s specifications. The enzymatic digestion was performed overnight at 37 °C, with slight shaking. Peptides were extracted from the gel pieces by a series of incubation steps (15 min each) using 50 mM ammonium bicarbonate, 100% acetonitrile, and 5% formic acid solution [75]. The collected solution was again dried under vacuum, dissolved in 1% formic acid, filtered through a 13.3 kDa spin-filter, and subsequently subjected to LC–MS/MS analysis.

### LC–MS/MS analysis, protein identification, and quantification

Protein analysis was performed using a timsTOF Pro mass spectrometer coupled with a NanoElute LC System (Bruker Daltonik GmbH, Bremen, Germany), equipped with an Aurora column (250 × 0.075 mm, 1.6 µm; IonOpticks, Hanover St., Australia). The mobile phase comprised a 0.1% (v/v) water–formic acid mixture (A) and a 0.1% (v/v) acetonitrile–formic acid mixture (B), which was added as a binary gradient at a flow rate of 0.4 µL/min. The gradient concentration started at 2% (v/v) B and was increased linearly to 17% B after 36 min. After another 18 min, the percentage of C was increased to 25% (v/v) and then increased linearly to 37% B after 6 min. After 70 min, the concentration of B was adjusted to the final value of 95% (v/v). The oven temperature during the measurements was 50 °C.

A timsTOF pro mass spectrometer (TIMS) was used in PASEF Mode with the following settings: mass range, 100–1700 mass: charge [m/z] ratio; ion mobility ramp, 0.6–1.6 V·s/cm^2^; 10 MS/MS scans per ion mobility ramp (total cycle time 1.16 s); charge range, 0–5; active exclusion for 0.4 min; a target intensity of 20,000 counts; and an intensity threshold of 1000 counts. Collision energy was ramped stepwise, appropriate to the ion-mobility ramp, from 20 to 59 eV. The electrospray ionisation source parameters were 1600 V for the capillary voltage and 3 L/min N_2_ (as dry gas) at a dry gas temperature of 180 °C. The measurements were performed in a positive ion mode. Mass calibration was performed using the sodium formate cluster, and the TIMS was calibrated using Hexais (2,2-difluoroethoxy) phosphazene, Hexakis (2,2,3,3-tetrafluoropropoxy) phosphazene, and Chip cube high mass references (m/z ratios of 622, 922, and 1222, respectively) [76, 77].

### Bioinformatics analysis

Peptide and subsequent protein identification were performed using PEAKS software (Bioinformatics Solutions Inc., Waterloo, ON, Canada) [78–80] and the *C. oleaginosus* protein sequences from the UniProt database [52]. The relative protein intensities were quantified as the ratio of protein intensities normalised to the total protein intensities from the respective sample, and three biologically independent measurements were performed. For each protein, a two-sample *t*-test was performed. Glycosylated peptides from the secreted fractions were identified using the available PMTs implemented in PEAKS software. Based on the identified protein sets (protein fractions and carbohydrates), an intersection analysis was performed to generate representative groups and Venn diagrams (http://www.interactivenn.net, as of 14.09.2020) (Additional files 13, 14).

The classification of the proteins into the Gene Ontology (GO) classes biological process, cellular function, and molecular function was performed using OmicsBox software (BioBam Bioinformatics S.L., Valencia, Spain, Version 1.3.11) and the identified protein sets. First, a BLAST search against the CloudBlast database and an Interpro scan on CloudIPS was performed. Then, the proteins were mapped and annotated before they were assigned to the respective classes. For a more precise functional analysis of the identified potential hydrolases, sequences of similar enzymes were identified using Swiss-Model software [81–85] and the UniProt database [52].

Phylogenetic analyses were performed in Geneious Prime® (v2021.1.1, Biomatters Ltd.) using the “muscle alignment” for the yeast 18 s sequences (American Type Culture Collection, Manassas, Virginia, USA) and the “geneious alignment” for the identified hydrolases using the “Geneious Alignment” with the consensus method majority greedy clustering.

### Identifying signal peptides targeting the protein-secretion pathway in *C. oleaginosus*

To identify potential eukaryotic signal peptides, the SignalP5.0 [62], Phobius [86], and WoLF PSORT [87] software packages were used. The extraction software was used to screen for secreted proteins with a -10 · lg_P_ higher than 36 and a signal sequence targeting the pathway of protein secretion. A hit was determined only when all three programs predicted a signal peptide for the protein.

MEME Suite 5.1.1 [88] was used for motif mining and identification in the selected dataset. Specifically, the MEME tool was used for novel motif prediction of ungapped motifs in the set of proteins potentially carrying a signal peptide. The FIMO tool was used to scan for certain motifs within a specific dataset.

## Supplementary Information

**Additional file 1: Fig. S1**. Phylogenetic comparison of the 18S ribosomal RNA genes from Cutaneontrichosporon oleaginosus, Trichosporon cutaneum, Trichosporon asahii, Rhodotorula toruloides, Yarrowia lipolytica, Lipomyces starkeyi, Komagataella pastoris and Saccharomyces cerevisiae.

**Additional file 2: Fig. S2**. Morphology of *C. oleaginosus* cultured in different carbohydrate-based media. The red scale bar represents 5 µm. In the left panel, microscopic images of yeast cells grown in different carbohydrate-based media are shown. *C. oleaginosus* cells cultured in (A) glucose, (B) cellobiose, (C) lactose, (D) maltose, (E) sucrose, and (F) trehalose are shown at 100× magnification. In the right panel, yeast cells cultured in the same carbohydrate-based media are shown following Nile red staining at 100× magnification.

**Additional file 3: Fig. S3**. Fatty acid content and profile of *C. oleaginosus* after cultivation on different disaccharides. Shown are the total fatty acid content (a) and the fatty acid profile of cultures grown for 5 days on different carbon sources and under nitrogen limitation.

**Additional file 4: Fig. S4**. Intersection analysis of the proteins identified in the different carbohydrate-based media. The Venn diagram shows the protein-intersection points of the investigated carbohydrates in relation to the identified proteins. The graph indicates, from top to bottom, the Venn diagrams for the secreted (a), cell wall-associated (b), and cytoplasmic fractions (c).

**Additional file 5: Fig. S5**. Classification of all identified proteins according to their functions in different bioprocesses.

**Additional file 6: Fig. S6**. Classification of all identified proteins according to their molecular functions.

**Additional file 7: Fig. S7**.Classification of all identified proteins according to their functions in different cellular processes.

**Additional file 8: Fig. S8**. Overview of the potential hydrolases identified in this study. The potential hydrolases identified in this study are listed, along with their identification (ID) numbers. The name describes the function of the enzyme with the highest sequence ID found after performing BLAST searches with the Swiss-model and UniProt databases. In addition, the reference ID is given for identification purposes. The Ref column indicates whether previous publications have identified the hydrolase of interest (+) or not (/).

**Additional file 9: Fig. S9**. Results of the enzyme test. Test results for each fraction for the enzymatic breakdown of the indicated carbohydrates are shown. Green highlighting indicates cleavage of the indicated disaccharide, whereas red highlighting indicates a negative reaction.

**Additional file 10: Fig. S10**. Overview of the most important hydrolases, as well as their function and potential substrates.

**Additional file 11: Fig. S11**. Phylogenetic analysis of the identified hydrolases. Major enzymes from figure EV10 are highlighted in red.

**Additional file 12: Table S1**. Total modified peptides.

**Additional file 13: Table S2**. Significants test of the detected hydrolases.

**Additional file 14: Table S3**. Total proteins detected. 

## Data Availability

All data generated or analysed during this study are included in this published article (and its Additional files).

## Abbreviations

LC-MS/MS: Liquid chromatography–tandem mass spectrometry
MW: Molecular weight
OD_600_: Optical density at 600 nm
TIMS: TimsTOF pro mass spectrometer
YNB: Yeast nitrogen base
