# Supplementary figures and images for: Identifying carbohydrate-active enzymes of Cutaneotrichosporon oleaginosus using systems biology
Source: Microb Cell Fact. 2021 Oct 28;20:205. doi: 10.1186/s12934-021-01692-2 (PMC8555327; doi:10.1186/s12934-021-01692-2)

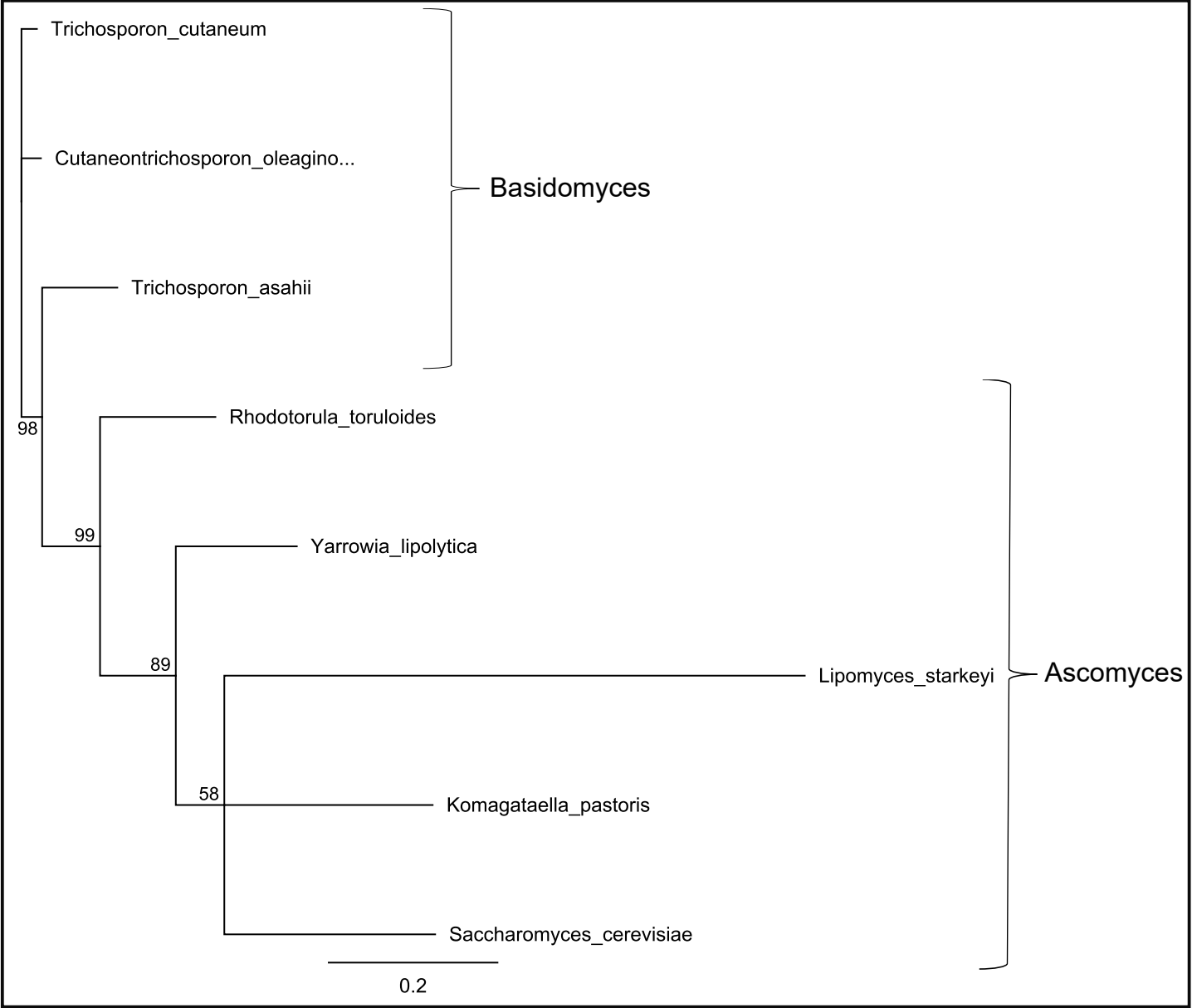

Supplement: Supplementary file 1 — Additional file 1: Fig. S1. Phylogenetic comparison of the 18S ribosomal RNA genes from Cutaneontrichosporon oleaginosus, Trichosporon cutaneum, Trichosporon asahii, Rhodotorula toruloides, Yarrowia lipolytica, Lipomyces starkeyi, Komagataella pastoris and Saccharomyces cerevisiae. [file 12934_2021_1692_MOESM1_ESM.pdf]

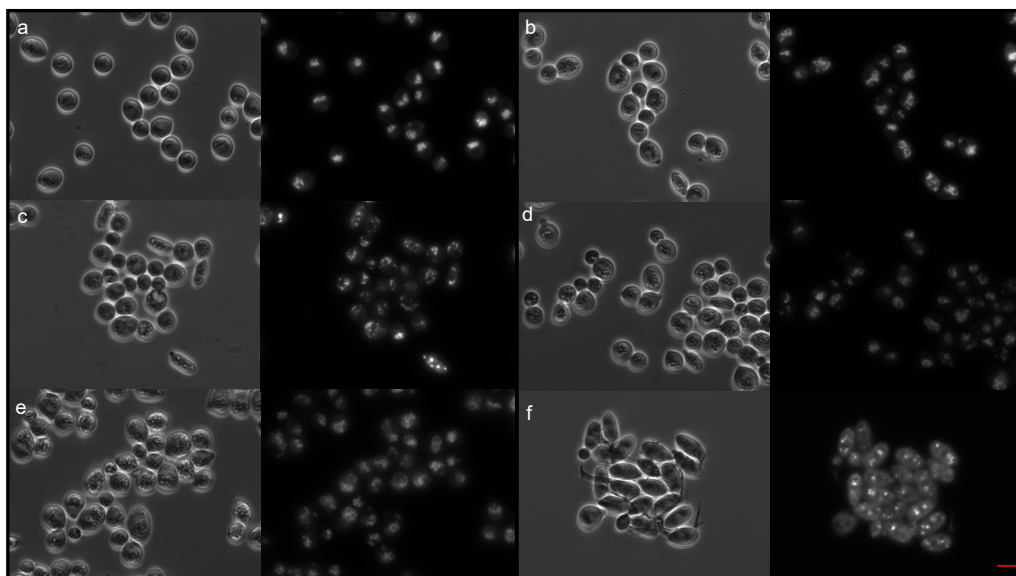

Supplement: Supplementary file 2 — Additional file 2: Fig. S2. Morphology of C. oleaginosus cultured in different carbohydrate-based media. The red scale bar represents 5 µm. In the left panel, microscopic images of yeast cells grown in different carbohydrate-based media are shown. C. oleaginosus cells cultured in (A) glucose, (B) cellobiose, (C) lactose, (D) maltose, (E) sucrose, and (F) trehalose are shown at 100× magnification. In the right panel, yeast cells cultured in the same carbohydrate-based media are shown following Nile red staining at 100× magnification. [file 12934_2021_1692_MOESM2_ESM.pdf]

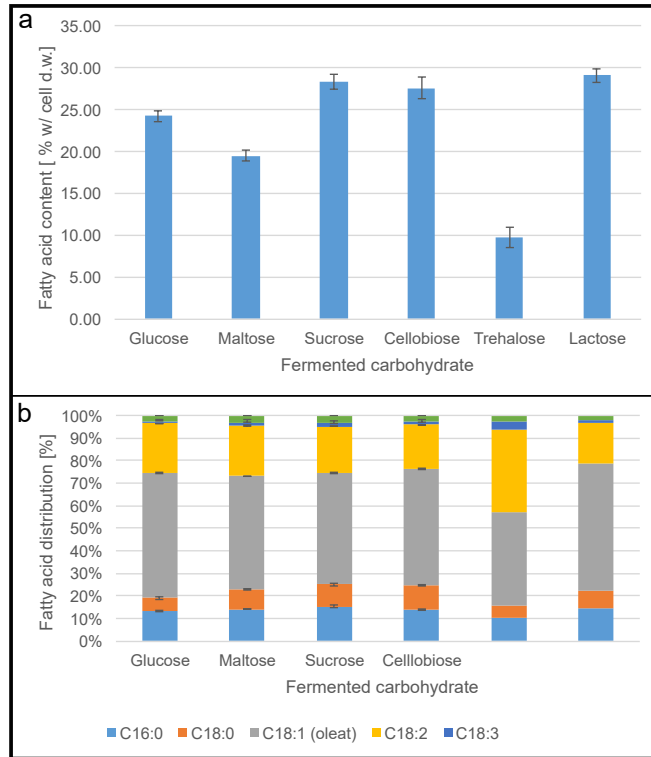

Supplement: Supplementary file 3 — Additional file 3: Fig. S3. Fatty acid content and profile of C. oleaginosus after cultivation on different disaccharides. Shown are the total fatty acid content (a) and the fatty acid profile of cultures grown for 5 days on different carbon sources and under nitrogen limitation. [file 12934_2021_1692_MOESM3_ESM.pdf]

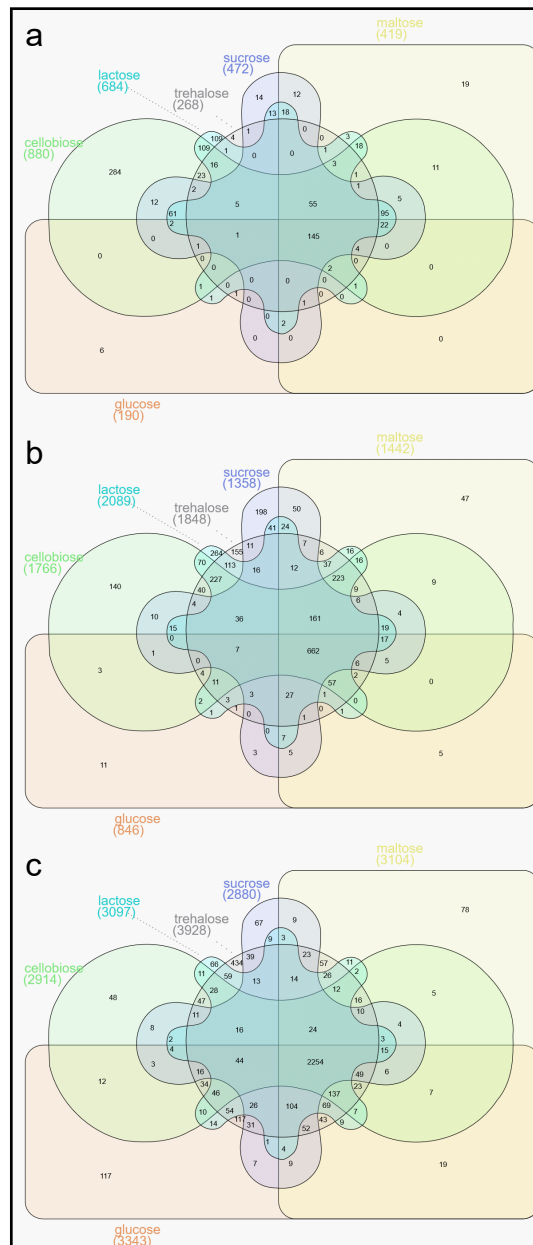

Supplement: Supplementary file 4 — Additional file 4: Fig. S4. Intersection analysis of the proteins identified in the different carbohydrate-based media. The Venn diagram shows the protein-intersection points of the investigated carbohydrates in relation to the identified proteins. The graph indicates, from top to bottom, the Venn diagrams for the secreted (a), cell wall-associated (b), and cytoplasmic fractions (c). [file 12934_2021_1692_MOESM4_ESM.pdf]

## secreted

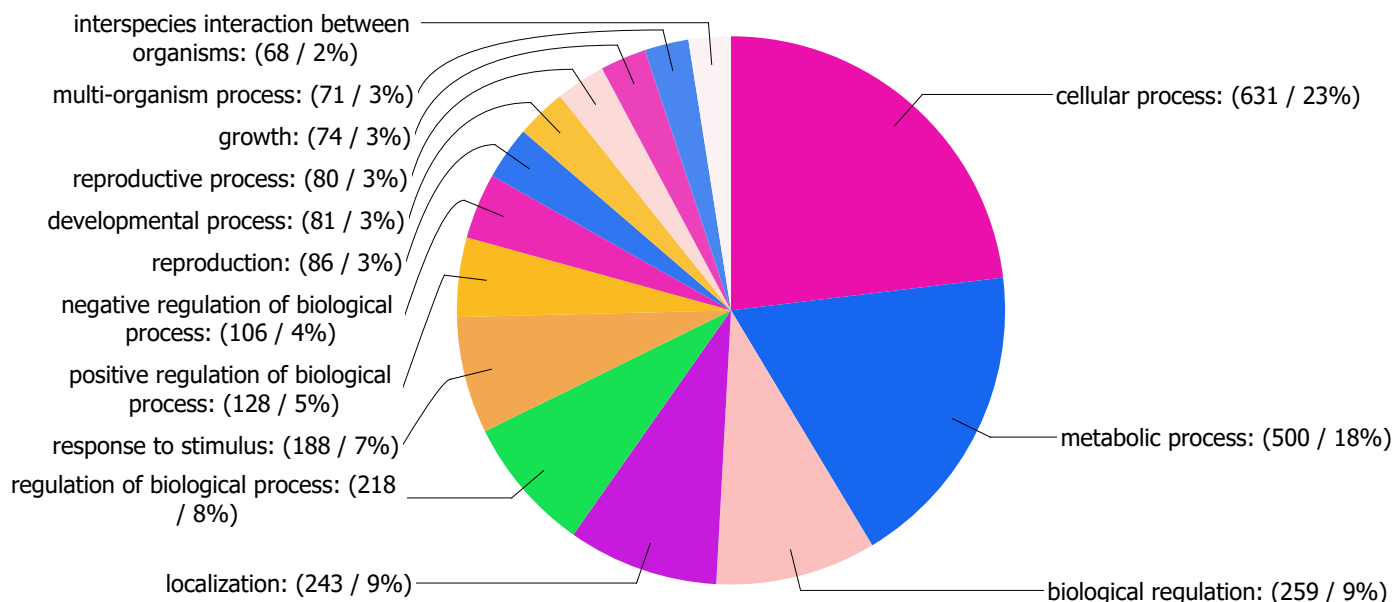

## cell wall-associated

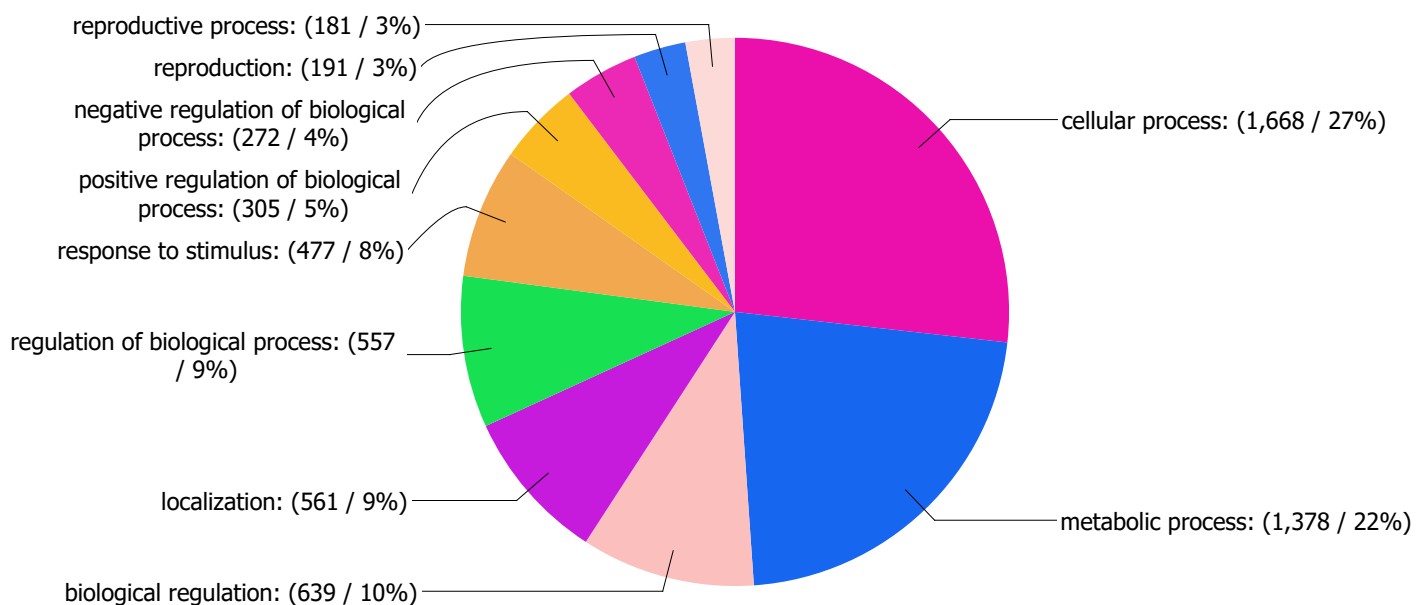

## cytoplasm

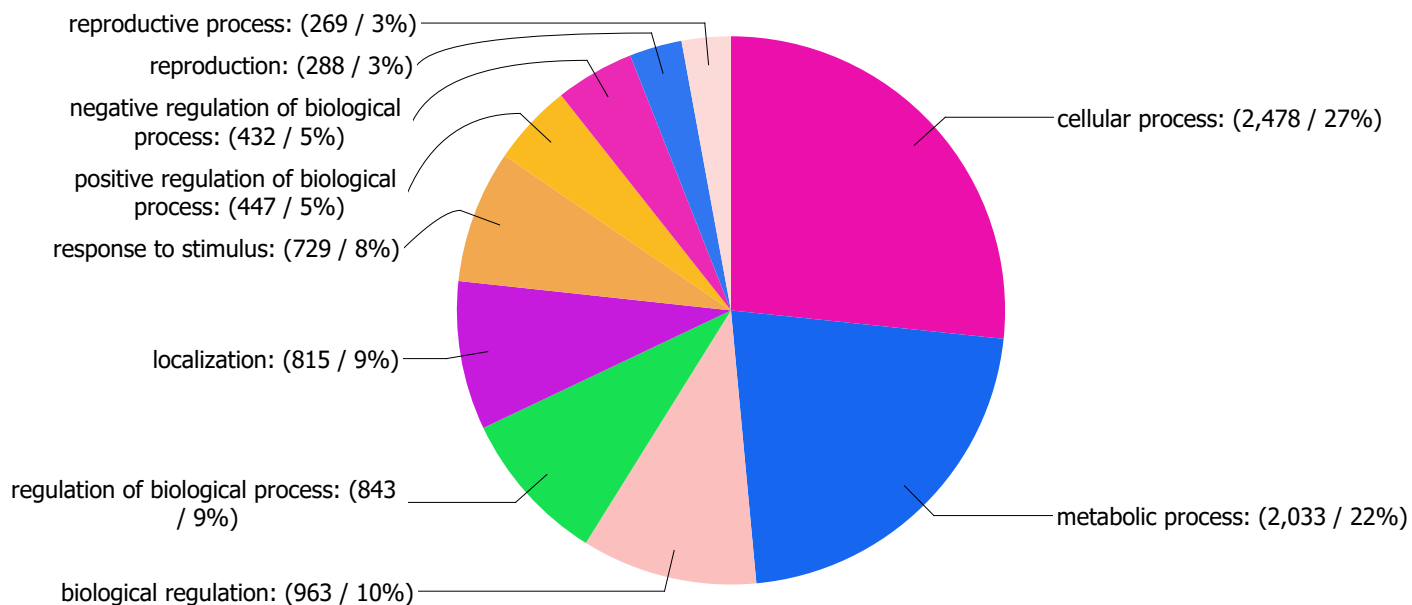

Supplement: Supplementary file 5 — Additional file 5: Fig. S5. Classification of all identified proteins according to their functions in different bioprocesses. [file 12934_2021_1692_MOESM5_ESM.pdf]

## secreted

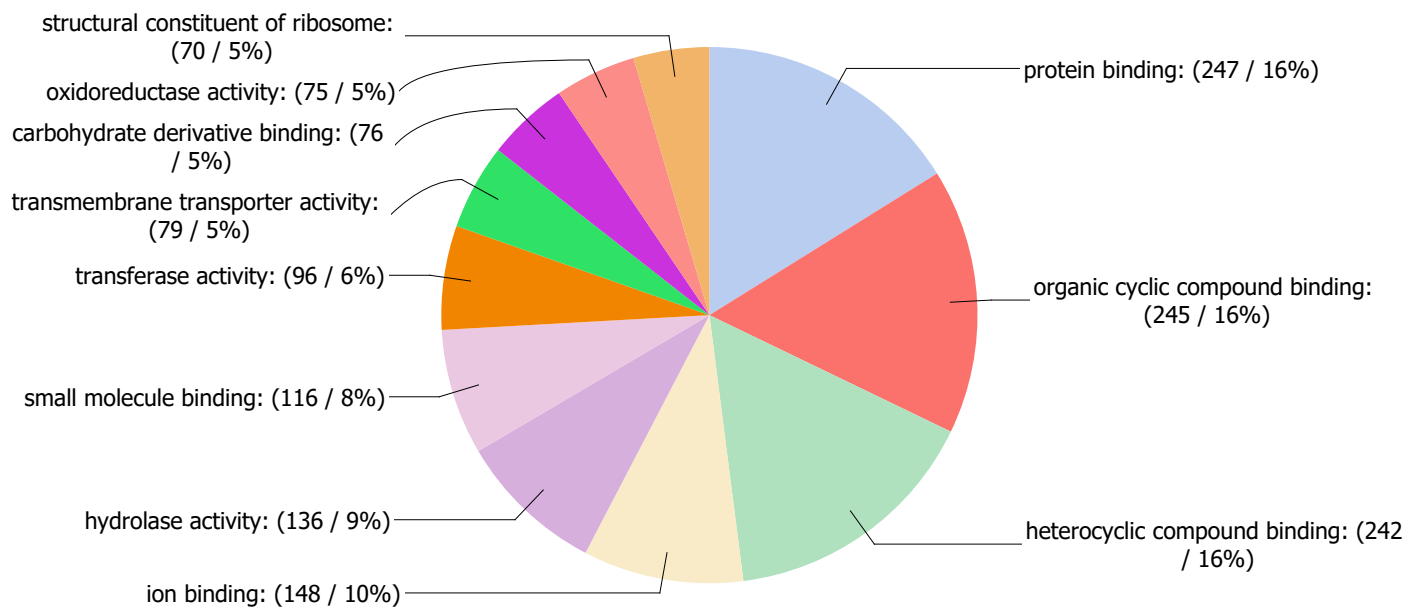

## cell wall-associated

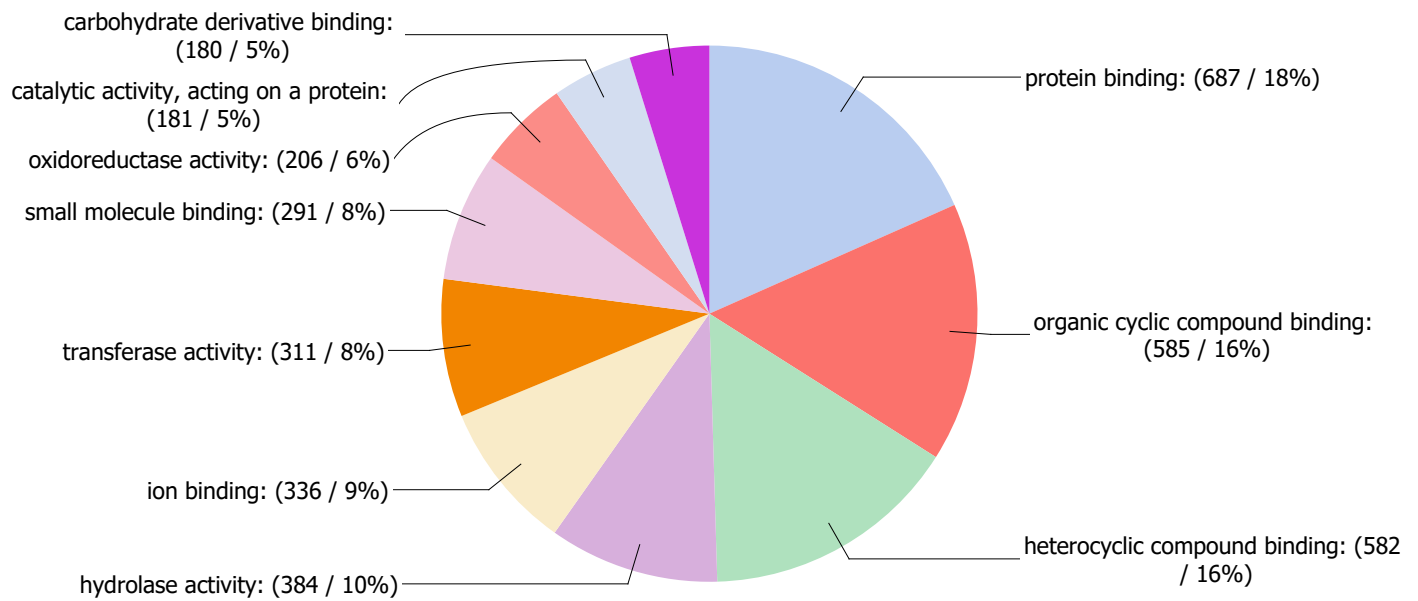

## cytoplasm

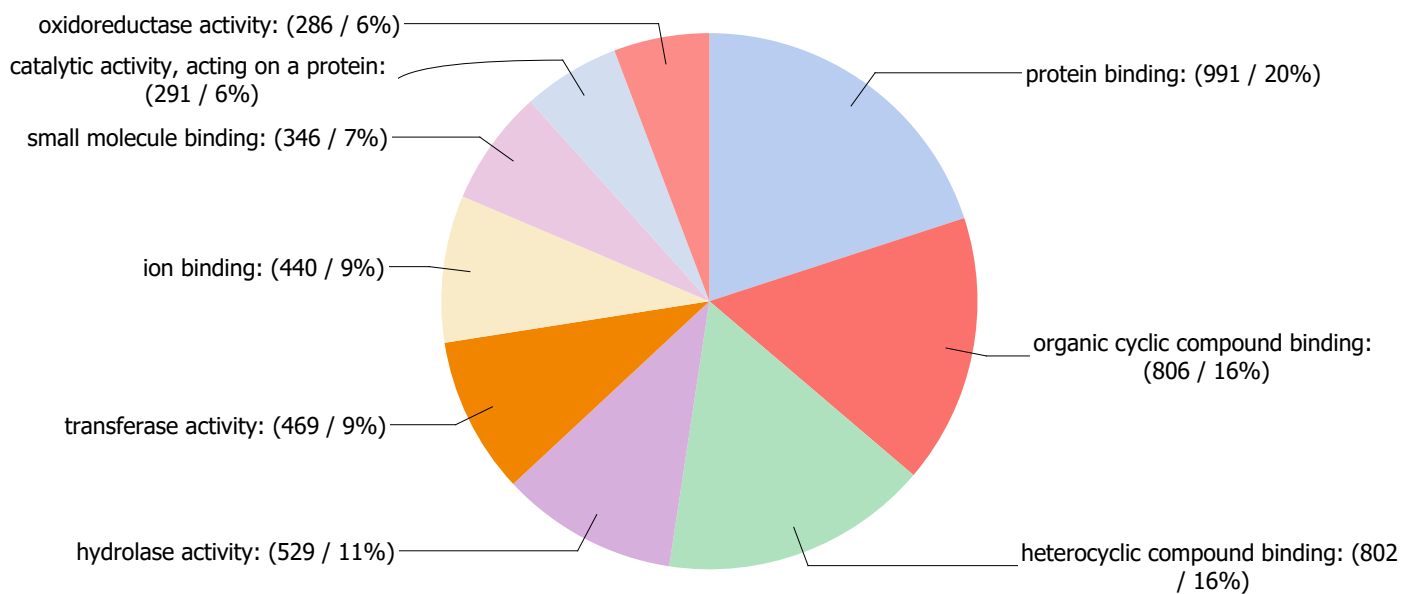

Supplement: Supplementary file 6 — Additional file 6: Fig. S6. Classification of all identified proteins according to their molecular functions. [file 12934_2021_1692_MOESM6_ESM.pdf]

### secreted

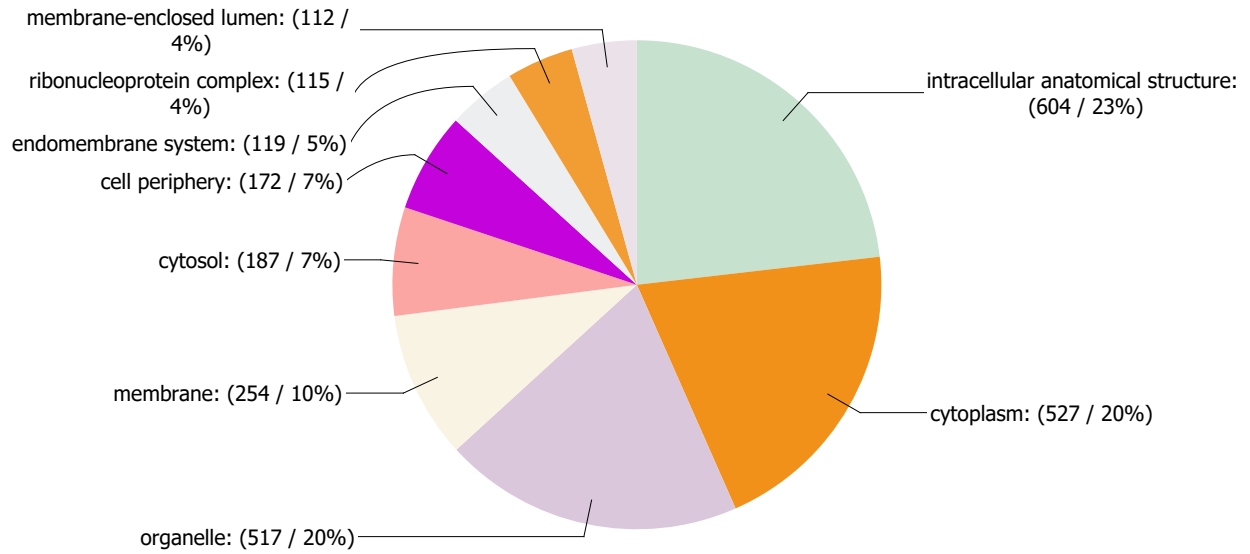

### cell wall-associated

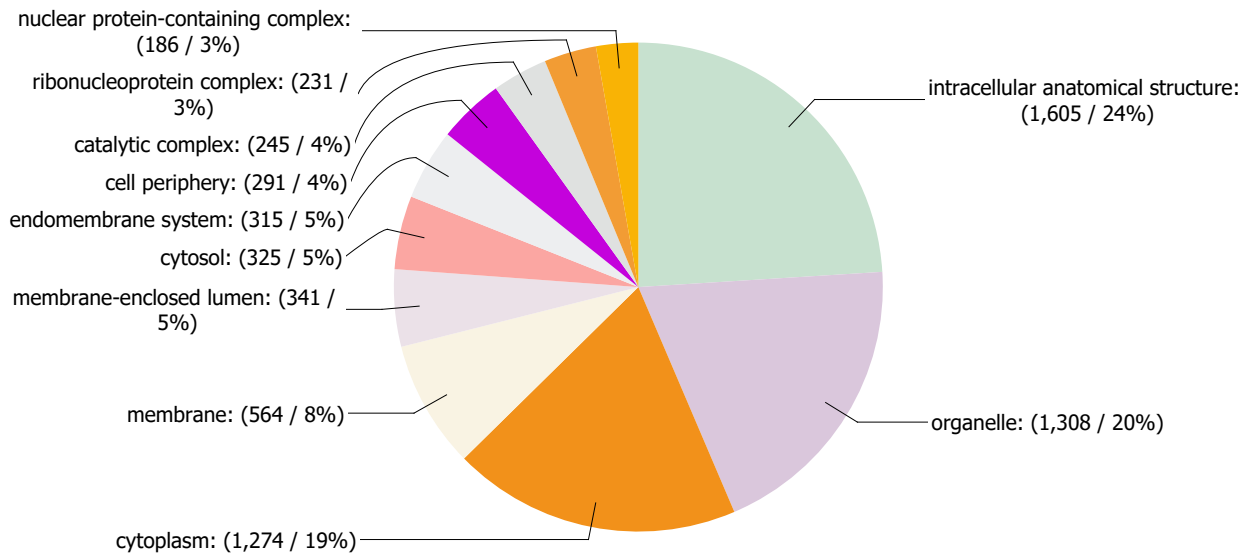

### cytoplasm

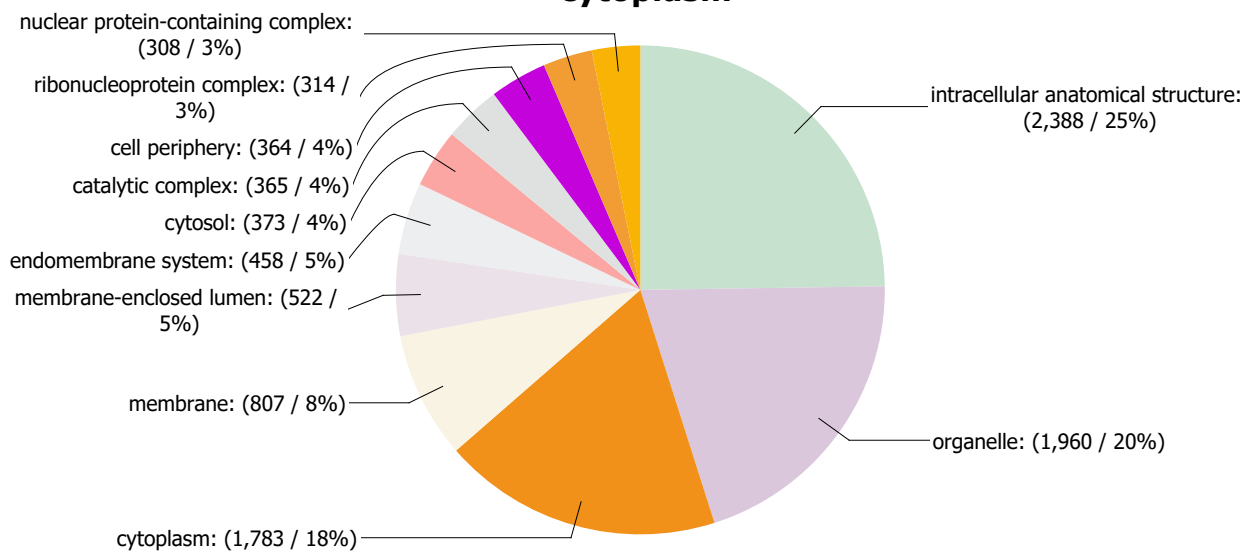

Supplement: Supplementary file 7 — Additional file 7: Fig. S7.Classification of all identified proteins according to their functions in different cellular processes. [file 12934_2021_1692_MOESM7_ESM.pdf]

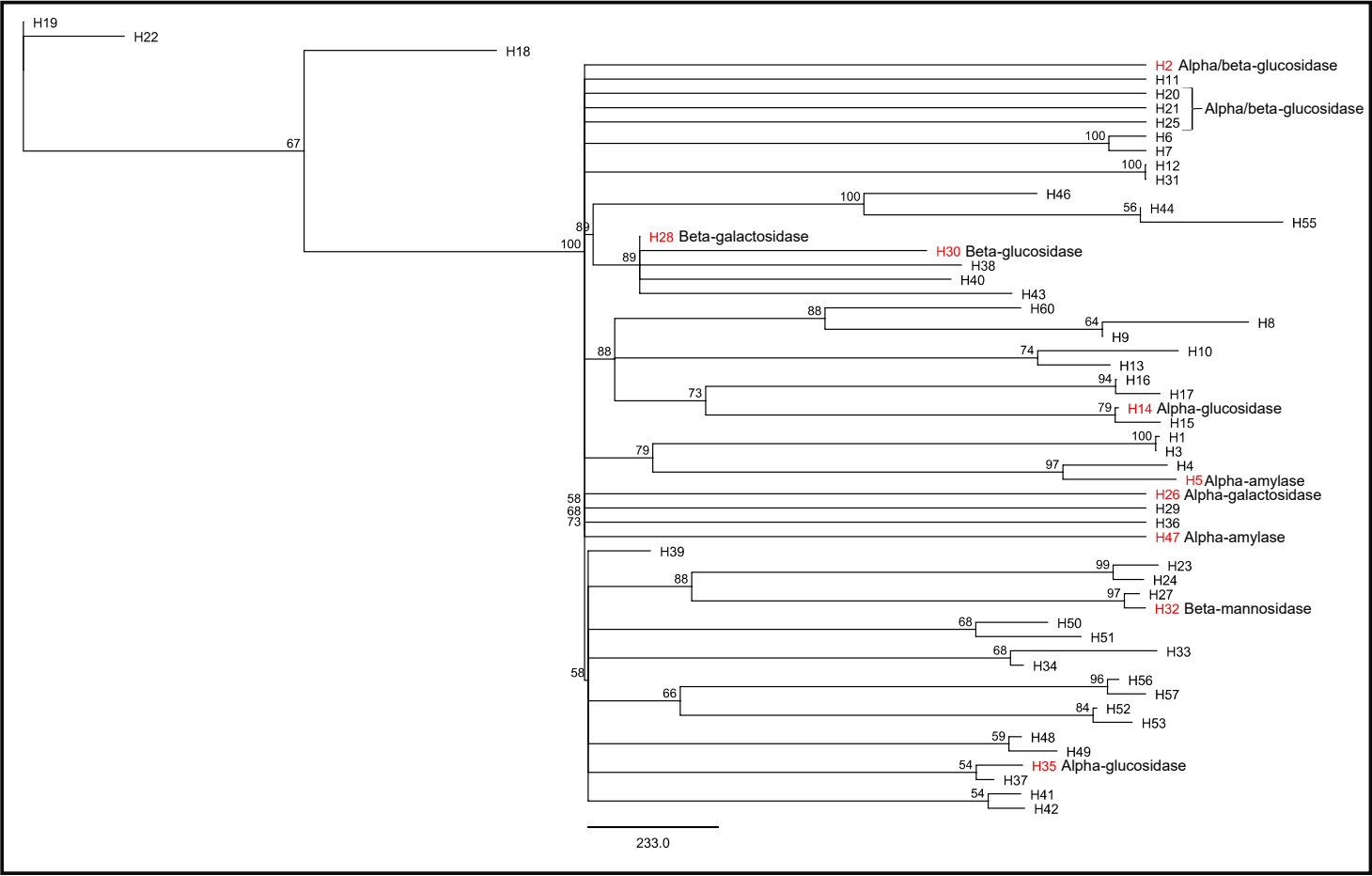

Supplement: Supplementary file 11 — Additional file 11: Fig. S11. Phylogenetic analysis of the identified hydrolases. Major enzymes from figure EV10 are highlighted in red. [file 12934_2021_1692_MOESM11_ESM.pdf]
