# Supplementary material for: Identifying carbohydrate-active enzymes of Cutaneotrichosporon oleaginosus using systems biology
Source: Microb Cell Fact. 2021 Oct 28;20:205. doi: 10.1186/s12934-021-01692-2 (PMC8555327; doi:10.1186/s12934-021-01692-2)
Supplement: Supplementary file 8 — Additional file 8: Fig. S8. Overview of the potential hydrolases identified in this study. The potential hydrolases identified in this study are listed, along with their identification (ID) numbers. The name describes the function of the enzyme with the highest sequence ID found after performing BLAST searches with the Swiss-model and UniProt databases. In addition, the reference ID is given for identification purposes. The Ref column indicates whether previous publications have identified the hydrolase of interest (+) or not (/). [file 12934_2021_1692_MOESM8_ESM.pdf]

NR	ID	Name/Function	Reference-ID	Sequence-ID [%]	Database	Ref.
H1	A0A0J1BEM6	Endo-1,3(4)-beta-glucanase	A0A1B9GRW3	57.0	Uniprot	/
H2	A0A0J0XHC0	Alpha/beta-glucosidase	J5R242	60.0	Uniprot	/
H3	A0A0J0XCY7	Invertase	A0A1B9GAJ1	35.7	Uniprot	/
H4	A0A0J0XDY7	Beta-1,3-1,4-glucanase	/	44.3	Swiss	+
H5	A0A0J0XT28	Alpha-Amylase	/	39.3	Swiss	+
H6	A0A0J0XNE6	Glucan endo-1,3-beta-glucosidase	Q5K792	56.3	Uniprot	/
H7	A0A0J0XJB9	Trehalase	A0A1E3I4Y3	52.5	Uniprot	/
H8	A0A0J0XB79	Dipeptidyl-peptidase 5	Q9P778	34.7	Uniprot	+
H9	A0A0J0XBX5	Endo-1,3(4)-beta-glucanase	A0A1E3IJ84	53.8	Uniprot	/
H10	A0A0J0XH28	Glucan endo-1,3-alpha-glucosidase agn1	O13716	38.3	Uniprot	+
H11	A0A0J0XWG8	1,4-alpha-glucan-branching enzyme	/	63.01	Swiss	+
H12	A0A0J0XM56	Exo-ß-(1,3)-glucanase	/	34.2	Swiss	+
H13	A0A0J1AV63	Chitinase	Q5KB41	51.1	Uniprot	/
H14	A0A0J1B7V0	Alpha-glucosidase	/	36.7	Swiss	+
H15	A0A0J1BBW0	EXO-B-(1,3)-GLUCANASE	/	42.5	Swiss	+
H16	A0A0J0XMG2	Chitin deacetylase	P82476	47.3	Uniprot	+
H17	A0A0J1B677	Glycoside hydrolase/deacetylase	A0A2H3BJE5	/	Uniprot	/
H18	A0A0J0XJI2	Endoglucanase	A0A427YAW0	57.3	Uniprot	/
H19	A0A0J0XSP4	Glucosidase	K1WV28	59.7	Uniprot	/
H20	A0A0J0XQV4	Abhydrolase	A0A427XIE1	59.5	Uniprot	/
H21	A0A0J1BBF9	Alpha/beta-hydrolase	A0A1Y2BJR1	57.3	Uniprot	/
H22	A0A0J0XCH8	Putative dienelactone hydrolase	/	19.7	Swiss	+
H23	A0A0J0XIP5	Alpha/beta-hydrolase	A0A1Y2AFQ6	51.5	Uniprot	/
H24	A0A0J0XDB8	Alpha/beta-hydrolase	A0A1Y2ATI6	57.9	Uniprot	/
H25	A0A0J0XZ69	Abhydrolase	A0A5M6C6U7	60.3	Uniprot	/
H26	A0A0J0XES7	Alpha-galactosidase	Q5AU92	54.9	Uniprot	+
H27	A0A0J0XGS2	Alpha-xylosidase	Q5AW25	47.8	Uniprot	+
H28	A0A0J0XCW3	Beta-galactosidase	/	45.8	Swiss	+
H29	A0A0J0XQT4	Beta-glucosidase	/	40.4	Swiss	+
H30	A0A0J1AWU3	Beta-glucosidase	Q25BW4	56.3	Uniprot	+
H31	A0A0J0XSL4	Glucan 1,3-beta-glucosidase	/	37.4	Swiss	+
H32	A0A0J0XLQ1	Beta-mannosidase	A0A427Y1C0	75.2	Uniprot	+
H33	A0A0J0XBA4	Glucocerebrosidase	H1AE12	58.5	Uniprot	/
H34	A0A0J0XQN3	Glycoside hydrolase	/	53.0	Swiss	+
H35	A0A0J1B1F9	Alpha-glucosidase	Q9P6J3	47.1	Uniprot	+
H36	A0A0J0XRK3	Uncharacterized protein	A0A427XTC5	43.9	Uniprot	/
H37	A0A0J1AUQ8	Uncharacterized protein	J6F857	64.1	Uniprot	/
H38	A0A0J0XGW6	Phosphate system	A0A427XF24	74.2	Uniprot	/
H39	A0A0J1ASG6	Chitin synthase	Q4P9K9	47.5	Uniprot	+
H40	A0A0J0XZM7	Dipeptidyl peptidase	/	34.6	Swiss	+
H41	A0A0J0XKP2	ATP_transf domain-containing	K1VQT8	57.7	Uniprot	/
H42	A0A0J1B5L2	Acylpyruvate hydrolase	A0A1B9H6E0	78.5	Uniprot	/
H43	A0A0J1AZB4	Putative metallocarboxypeptidase	B6H233	33.8	Uniprot	+
H44	A0A0J0XIC5	Cleavage and polyadenylation specificity factor	A0A427YBW7	69.2	Uniprot	/
H46	A0A0J0XHA3	Alpha glucosidase	/	44.6	Swiss	+
H47	A0A0J1BA01	alpha-amylase	/	48.1	Swiss	+
H48	A0A0J0XBA7	Beta-galactosidase	/	45.8	Swiss	+
H49	A0A0J0XV52	UDP-glucose 4-epimerase	/	48.1	Swiss	+
H50	A0A0J0XC30	Neutral trehalase	/	50.1	Swiss	+
H51	A0A0J0XYB4	Alpha/beta-hydrolase	A0A1Y2BE77	32.2	Uniprot	/
H52	A0A0J0XN97	Glycosyl hydrolase	A0A1Y1UM13	46.9	Uniprot	/
H53	A0A0J0XGU5	Glycoside hydrolase family 16 protein	A0A427Y492	58.5	Uniprot	/
H55	A0A0J0XV92	Abhydrolase	A0A427YBU7	68.7	Uniprot	/
H56	A0A0J0XU26	Abhydrolase	A0A427Y9S0	54.1	Uniprot	/
H57	A0A0J0XIZ6	alpha-1 ,2-Mannosidase	/	43.3	Swiss	+
H60	A0A0J1BAM0	Alpha-L-AF_C domain-containing	A0A427XKP4	77.3	Uniprot	+
