# Supplementary material for: Identifying carbohydrate-active enzymes of Cutaneotrichosporon oleaginosus using systems biology
Source: Microb Cell Fact. 2021 Oct 28;20:205. doi: 10.1186/s12934-021-01692-2 (PMC8555327; doi:10.1186/s12934-021-01692-2)
Supplement: Supplementary file 10 — Additional file 10: Fig. S10. Overview of the most important hydrolases, as well as their function and potential substrates. [file 12934_2021_1692_MOESM10_ESM.pdf]

Protein ID	Name	Function	Possible substrates
H2	Alpha/beta-glucosidase	Cleveas 1,4-α-glucosidic linkages as well as β 1-4 linkages compounds	Maltose, Cellobiose, Glycolipids
H5, H47	Alpha-amylase	Cleaves α(1-4)-glycosidic compounds of amylose	Starch from maize, wheat or potato
H14, H35	Alpha-glucosidase	Cleavage 1,4-α-glucosidic compounds	Maltose
H26	Alpha-galactosidase	Catalyses the hydrolysis ofα-galactopyranosides	Glycosphingolipids, Raffinose, stachyose and verbascose
H28	Beta-galactosidase	Cleaves terminal-boundβ-D-galactose from molecules	Lactose, Gangliosides, glycoproteins and glycosaminoglycans
H30	Beta-glucosidase	Hydrolyseβ-glycosidic bonds with glucose	Cellobiose, Glycolipids
H32	Beta-mannosidase	Hydrolyses β-D-mannose residues into beta-D-mannosides	Mannan from Guar gum, carob or brown algae
